# Modeling and controlling a half-car model suspension system using an ANFIS controller

**DOI:** 10.1371/journal.pone.0361999

**Published:** 2026-11-05

**Authors:** Sisay Ewunetie Fisha, Amanuel Gebisa Aga, Tatek Mamo Wachamo

**Affiliations:** 1 Department of Motor Vehicle Engineering, Engineering College, Ethiopian Defence University, Bishoftu, Ethiopia; 2 Department of Mechanical Engineering, Center of Excellence for Transport and Vehicle Engineering, Adama Science and Technology University, Adama, Ethiopia; National University of Singapore, SINGAPORE

## Abstract

Passive suspension systems (PSS) use fixed spring and damping characteristics and cannot adapt to changing road disturbances and operating conditions. Active suspension systems (ASS) address this limitation by generating additional control forces, but controller design must balance ride comfort, road holding, model uncertainty, and control effort. This study develops a four-degree-of-freedom (4-DOF) longitudinal half-car model and evaluates an Adaptive Neuro-Fuzzy Inference System (ANFIS) controller against PSS and PID-controlled ASS under double-bump and ISO-classified random Type A and Type C Road profiles at 10 and 20 m/s. The ANFIS controller is trained using PID-generated input-output data with two inputs, five Gaussian membership functions per input, 25 Takagi-Sugeno rules, and 150 training epochs. For the double-bump case at 10 m/s, ANFIS reduces body vertical displacement from 0.0435 to 0.0084 m (80.6%), vertical acceleration from 2.40 to 0.77 m/s^2^ (67.9%), and front suspension travel from 0.0652 to 0.0090 m (86.2%) relative to PSS. Compared with PID, the corresponding reductions are 53.6%, 49.3%, and 43.8%, respectively. Settling time is reduced by 38.8% for body vertical displacement and 38.6% for pitch acceleration relative to PSS. The result meets all the qualifications for riding comfort of the ISO 2631 standard. The study identifies limitations related to PID-supervised training, linear modeling, and actuator-energy assessment by employing simulation. Experimental evaluation is required to establish robustness to parameter uncertainty, sensor noise, actuator delay, and implementation energy requirements.

## 1. Introduction

Among the critical subsystems that define a vehicle’s dynamic behavior, the suspension system plays a central role in ensuring ride comfort, road handling stability, and safety by softening the transmission of road-induced vibrations to the vehicle body and its occupants [1]. The vehicle’s suspension system minimises the transfer of static and dynamic forces between the vehicle and the road. It consists of parts that associate with each other to isolate the body of the vehicle from the wheels. The suspension system most often consists of three distinctive parts: first, there is a structure that holds the weight of the vehicle and defines the geometry of the suspension; then there is the spring, which can store energy and release it; finally, there is the shock absorber, which dissipates kinetic energy [2]. The suspension system connects the vehicle’s sprung mass (comprising the chassis, body, engine, and passengers) to the unsprung mass (wheels, axles, and brakes) through springs, dampers, and linkages [3].

Suspension systems are generally classified into three primary categories: passive suspension systems (PSS), semi-active suspension systems (SASS), and active suspension systems (ASS). The PSSs have fixed spring and damping characteristics, which hinder their adaptability to dynamic driving and road conditions [4]. PSS are simple, reliable, and cost-effective, involve a compromise between ride comfort and handling performance, and their parameters cannot be adjusted in real time. The SASS is an advanced version of vehicle suspension, which provides an appropriate variation in the real-time damping characteristics without the invasion of external energy into the system, as ASSs do. Typically, it uses controllable dampers such as Electro-Rheological (ER) or Magneto-Rheological (MR) devices whose damping force may be modulated by electrical signals, such as pulse-width modulation. The SASS achieves a good compromise between the simplicity and reliability of PSSs and the performance benefits of ASSs. They have low power consumption, have inherent fail-safety, and greatly improve ride comfort and road handling by adapting to changing road conditions and driving dynamics [5].

The ASS incorporating hydraulic, pneumatic, or electromagnetic actuators that generate real-time control forces to counteract road disturbances [6]. These systems offer superior performance by dynamically adjusting suspension behavior based on sensor feedback, thereby improving both comfort and stability [7]. However, the design of an effective controller for ASS remains a significant challenge due to the nonlinear, time-varying, and uncertain nature of vehicle-road interactions. Vehicle dynamics are commonly modeled using simplified representations such as the quarter-car, half-car, bicycle-car, or full-car models, each capturing different degrees of motion [8]. The longitudinal half-vehicle representation is particularly effective for studying pitch motion during acceleration, braking, or traversal over speed bumps, as it includes front and rear unsprung masses along with heave and pitch dynamics of the sprung mass.

Recent research has increasingly adopted Adaptive Neuro-Fuzzy Inference Systems (ANFIS) for suspension control because they combine fuzzy-rule interpretability with data-driven parameter adaptation. For example, Gandhi et al. compared PID, LQR, fuzzy, and ANFIS controllers for a 4-DOF half-car model and identified ANFIS as the strongest performer, although with increased actuator effort [9]. More recently, Seyyed Esmaeili et al. trained ANFIS using data from H2 and H∞ controllers and reported 62% improvement in body comfort and 57% improvement in ride safety relative to passive suspension for a quarter-car model [10]. These results support the potential of ANFIS, but they also show that reported gains depend strongly on the model, training data, road excitation, and performance metric. The present study extends this line of work to a 4-DOF half-car representation that explicitly includes sprung-body heave and pitch together with front and rear unsprung motions and evaluates both deterministic double-bump and random road profiles.

Recent developments in robust and intelligent suspension control further emphasize the need to account for uncertainty. Emami et al. proposed a genetic-algorithm-optimized fuzzy sliding-mode controller for a nonlinear full-vehicle semi-active suspension while explicitly considering sprung-mass variation, temperature effects, and vehicle ageing [11]. Najafi et al. developed a multidimensional fuzzy sliding-mode controller for a series active variable-geometry suspension and demonstrated reductions in bounce, pitch, roll acceleration, and tire deflection [12]. These approaches are more explicitly robust than the present ANFIS formulation. Their advantage is disturbance and uncertainty handling through robust control structure, whereas the present approach emphasizes a comparatively simple data-driven controller that can be implemented from input-output suspension data without requiring an online solution of a constrained optimization problem.

Hybrid controllers such as HANFISPIDCR and HFPIDCR have also demonstrated improved vibration suppression in simulation [13]. However, their hybrid structure increases controller complexity and can retain dependence on the assumptions and tuning of the underlying PID/fuzzy components. The present ANFIS controller is intentionally structured as a single nonlinear mapping from tracking error and error rate to actuator force. Its technical rationale is therefore not that ANFIS is universally superior to hybrid control, but that a compact learned nonlinear control law may provide a favorable balance between implementation simplicity and adaptability for the 4-DOF half-car problem. This distinction is important because the available results do not establish a general superiority of ANFIS over HANFISPIDCR or HFPIDCR [14].

Active suspension research is also moving toward coordinated chassis control. Liang et al. developed a decentralized cooperative framework integrating active steering and active suspension using multi-constrained distributed model predictive control, with simulation and hardware-in-the-loop validation [15]. Feng et al. subsequently integrated torque vectoring and active suspension using a linear-time-varying half-vehicle model and an MPC formulation that jointly considered safety, energy saving, and comfort, with rapid-control-prototype validation [16]. Liang et al. further proposed a game-theory-based multi-agent framework for coordinating torque vectoring and active suspension in distributed-drive electric vehicles, again using a half-vehicle model and experimental validation [17]. These studies illustrate the rapid development of active suspension from stand-alone vibration control toward integrated chassis control. The present work addresses a narrower but important problem: evaluating a computationally simple ANFIS controller for the coupled heave-pitch dynamics of a conventional 4-DOF half-car model across several road conditions.

The performance of suspension components also depends on the trade-off among spring stiffness, damping, tire stiffness, and suspension travel [18]. Existing half-car studies have investigated fuzzy control [19], LQR [20], PID [21], and hybrid approaches under simulated road disturbances [14], while comparative work has shown the potential of ANFIS for half-car vibration suppression [22]. Nevertheless, three gaps remain relevant to the present study. First, many reported ANFIS applications are based on quarter-car models or controller-specific datasets, which do not explicitly capture the front-rear coupling associated with pitch. Second, PID-generated training data can transfer the strengths and weaknesses of the baseline controller into the learned control law. Third, many simulation studies report performance improvements without explicitly separating deterministic effect size from statistical uncertainty or evaluating actuator effort and robustness to model and measurement uncertainties.

Accordingly, this study aims to model and control a 4-DOF half-car suspension system using ANFIS to compare it with PSS and PID-controlled ASS under double-bump and ISO Type A and Type C random road profiles. The study focused on body heave and pitch responses, front and rear suspension travel, and settling behavior. The contribution is a systematic simulation comparison of a compact ANFIS control law under both transient and stochastic road excitation, together with a critical assessment of PID-supervised training and deterministic simulation.

The remaining sections of this study is organized as follows. Section 2 presents materials and methods which included the vehicle parameters, half-car mathematical model, state-space representation, ANFIS architecture and training procedure, and road profiles. Section 3 presents the simulation results for the double-bump and random road inputs and discusses their physical meaning, including the implications of controller effort and robustness. Section 4 concludes the main findings and identifies the limitations, and experimental validation needs of the present study.

## 2. Materials and methods

This study follows a systematic approach to achieve its aim. First, a thorough literature survey was performed. Then, mathematical models for both the ASS and the PSS were developed for comparison. Next, an ANFIS controller has been designed for the 4 DoF half-car model and implemented in MATLAB/Simulink. Finally, the performance of the system was compared for ASS and PSS using key parameters. The study confirmed the effectiveness of the proposed system, as the results show significant reductions in body and pitch displacements, and front and rear wheel vibrations.

The study considers a 2017 compact hatchback vehicle commonly used in urban driving, characterized by its lightweight and agile nature. The parameters in Table 1 were adopted from the published half-car vehicle model used as the source for the Honda Civic 2017 representation [18]. Because the present study is simulation-based, these values were not independently identified from vehicle test measurements. As an internal consistency check, the front and rear distances from the center of gravity sum to the stated wheelbase (1.015 + 1.895 = 2.910 m). The remaining mass, stiffness, damping, tire-stiffness, and pitch-inertia values were retained from the cited model so that the present controller comparison is reproducible. Nevertheless, these parameters are subject to uncertainty arising from payload, tire pressure, temperature, component ageing, and manufacturing variation. Such uncertainty can change the natural frequencies, damping ratios, and actuator demand of the model. Because a systematic sensitivity study was not the focus of this study, the performance improvements reported below should be interpreted as nominal-model or simulation results rather than as experimental validation.

**Table 1:** Technical Specifications of Honda Civic 2017 model [18].

Description	Value	Unit
Sprung mass ($m_{s}$)	635	kg
Front unsprung masses ($m_{uf}$)	35.5	kg
Rear unsprung masses ($m_{ur}$)	35.5	kg
Front suspension spring stiffness ($k_{sf}$)	27000	N/m
Rear suspension spring stiffness ($k_{sr}$)	30000	N/m
Front spring tire stiffness ($k_{uf}$)	268000	N/m
Rear spring tire stiffness ($k_{ur}$)	268000	N/m
Front suspension damping coefficient ($c_{sf}$)	2570	Ns/m
Rear suspension damping coefficient ($c_{sr}$)	2570	Ns/m
Front suspension length from CG ($L_{r}$)	1.015	m
Rear suspension length from CG ($l_{f}$)	1.895	m
Wheelbase (L)	2.91	m
Moment of Inertia about pitch axis (J)	768.35	$\frac{kg}{m^{\text{2}}}$
Front wheel weights (Wf)	1434.65	N
Rear wheel weights (Wr)	2376.54	N

### 2.1. Mathematical modeling of a half-car model

This section develops mathematical models of both ASS and PSS for comparative analysis. Accordingly, the governing equations of the vehicle model under dynamic conditions are formulated. The free body diagram of the half-car vehicle model for an ASS used in this study is given in Fig 1. The sprung mass (m_s_) refers to the portion of the vehicle consisting of the body, chassis, and internal components that are supported by the suspension system. The unsprung masses at the front and rear (m_uf_ and m_ur_, respectively) correspond to the masses of the wheel assemblies and axles. The front and rear suspension spring stiffnesses are denoted as k_sf_ and k_sr_, while c_sf_ and c_sr_ represent the damping coefficients of the front and rear suspensions, respectively. The tire stiffness parameters for the front and rear are given as k_uf_ and k_ur_. Additionally, f_sf_ and f_sr_ denote the actuator-generated forces applied at the front and rear suspensions, respectively. The w_sf_ and w_sr_ are the front and rear road disturbance inputs acting on the unsprung mass, respectively, and θ is the vehicle rotational displacement, xs is the sprung mass vertical displacement, x_uf_ and x_ur_ are the front and rear unsprung mass vehicle vertical displacement. and l_f_ and l_r_ are the front suspension length from CG and the rear suspension length from CG, respectively.

**Fig 1:**
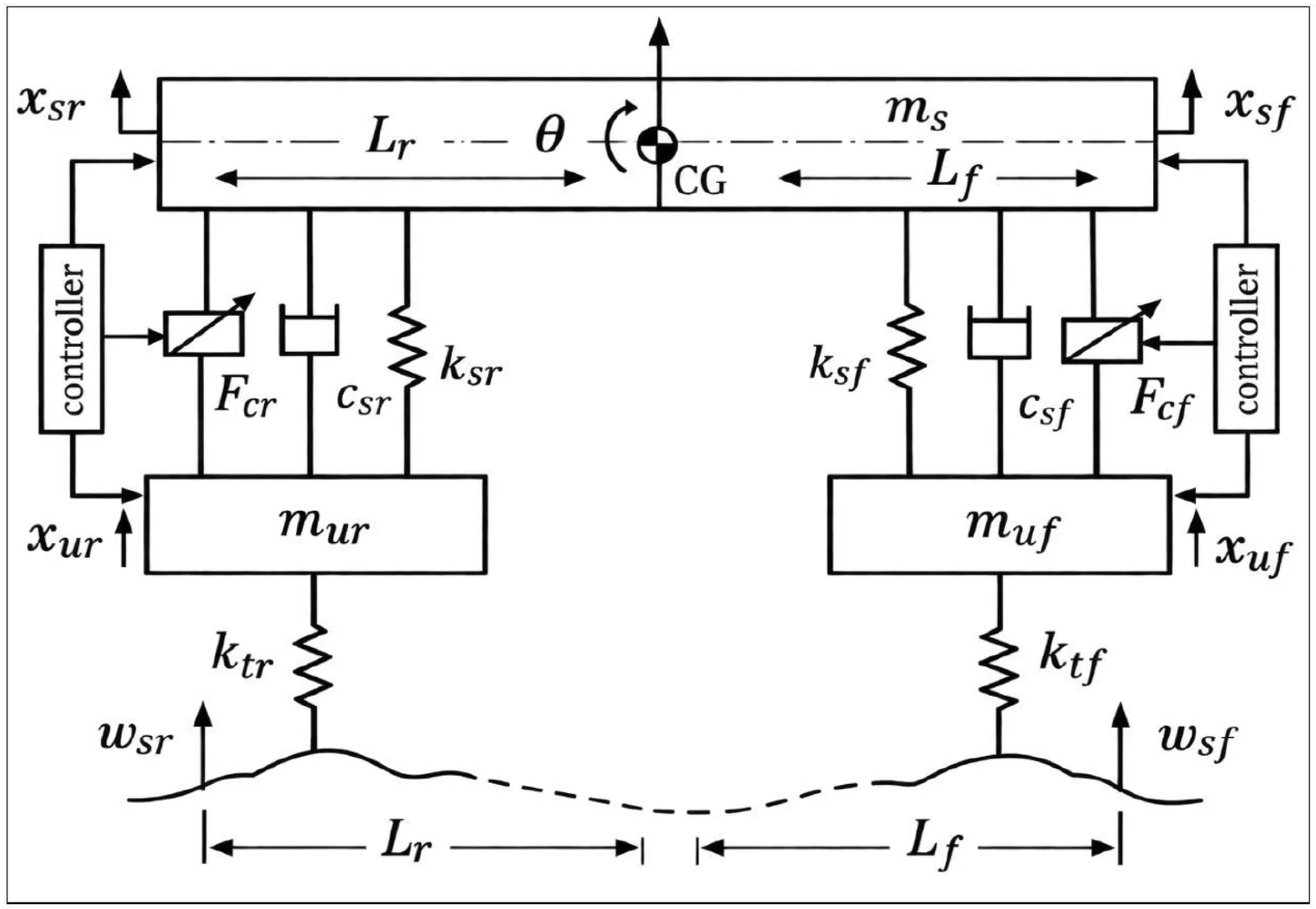
4-DOF half car model with controller.

Although real vehicle suspension dynamics are nonlinear because of tire-road contact, suspension nonlinearities, friction, actuator saturation, and parameter variation, a linear 4-DOF model is used here for a controlled comparison of PSS, PID-ASS, and ANFIS-ASS under moderate vertical disturbances. The linear formulation preserves the essential coupled heave-pitch and front/rear unsprung dynamics while keeping the plant computationally transparent and consistent with the available parameter set. This choice is also consistent with recent integrated active-suspension studies that employ linear or linear-time-varying half-vehicle models to reduce controller-design complexity [23]. The limitation is that the model does not reproduce nonlinear tire-force saturation, suspension bump-stop effects, friction, or large-deflection behavior. Therefore, the ANFIS controller should not be interpreted as having been validated for strongly nonlinear operating regimes.

In this study, the Euler- Lagrange formulation was used to develop a half-car model. In this approach, the system dynamics arise from the energy-based view by taking the Lagrangian function to be the difference between the total kinetic and potential energy. The resulting equations reproduce the standard four-DOF longitudinal half-car structure consisting of sprung-body heave, sprung-body pitch, and front and rear unsprung heave motions. The same state variables and force pathways are used in established half-car formulations and comparative studies [24]. Thus, the derived equations were checked structurally against the accepted half-car representation rather than treated as a new vehicle-dynamics formulation. Since no independent experimental identification was conducted, this verification supports model formulation consistency but does not constitute experimental validation. This formulation allows systematic derivation of the governing equations of motion by making use of the suitable generalized coordinates and forces as given in equation (1).


$$\begin{array}{c} L=K-V \end{array}$$ (1)

Where 𝐿 donates the Lagrangian of the system, K represents the kinetic energy, and V stands for potential energy.

Based on this function, the Lagrange equation of motion for a mechanical system with n degrees of freedom (DOF) can be derived as presented in equation (2) [25].


$$\begin{array}{c} \frac{d}{dt}\left(\frac{\partial K}{\partial \dot{X}}\right)-\frac{\partial K}{\partial X}+\frac{\partial D}{\partial \dot{X}}+\frac{\partial V}{\partial x}=f \end{array}$$ (2)

Where K is kinetic energy, D is dissipation energy, V is potential energy, f is actuator force, X is independent variables (like distances or angles), and $\dot{X}$ is velocity.

The total kinetic energy (K) is obtained by summing the translational energies of the sprung and unsprung masses with the rotational energy of the sprung mass, as given in equation (3).


$$\begin{array}{c} K=\frac{\text{1}}{\text{2}}J{\dot{\theta }}^{\text{2}}+\frac{\text{1}}{\text{2}}m_{s}{{\dot{x}}^{\text{2}}}_{s}+\frac{\text{1}}{\text{2}}m_{uf}{{\dot{x}}^{\text{2}}}_{uf}+\frac{\text{1}}{\text{2}}m_{ur}{{\dot{x}}^{\text{2}}}_{ur} \end{array}$$ (3)

Where K is kinetic energy, J is the moment of inertia about the pitch axis,$m_{s}$ is a sprung mass,$m_{uf}$ is the front unsprung mass,$m_{ur}$ is the rear unsprung mass,$\dot{\theta }$ is pitch angular velocity,$\dot{x_{s}}$ is the vertical velocity of the sprung mass,$\dot{x_{uf}}$ is the front unsprung mass velocities and $\dot{x_{ur}}$ is the rear unsprung mass velocities.

The total potential energy (V) is the sum of the vehicle body’s translational and rotational energies and the translational energies of the front and rear wheels, as expressed in equation (4).


$$\begin{array}{c} V=\frac{\text{1}}{\text{2}}k_{sf}(x_{s}+L_{f}\theta -x_{uf}{)}^{\text{2}}+\frac{\text{1}}{\text{2}}k_{sr}(x_{s}-L_{r}\theta -x_{ur}{)}^{\text{2}}+\frac{\text{1}}{\text{2}}k_{uf}(x_{uf}-w_{sf}{)}^{\text{2}}+\frac{\text{1}}{\text{2}}k_{ur}(x_{ur}-w_{sr}{)}^{\text{2}} \end{array}$$ (4)

Where V is potential energy, $k_{sf}$ is the front suspension spring stiffness, $k_{sr}$ is Rear suspension spring stiffness, $k_{tf}$ is Front tire stiffness, $k_{tr}\;$is Rear tire stiffness, $x_{s}$ is Vertical displacement of sprung mass, θ is Pitch angle,${\;L}_{f}$ Distance from CG to front axle,${\;L}_{r}$ Distance from CG to rear axle,$\;x_{uf}$ Front unsprung displacement, $x_{ur}$ is rear unsprung displacement,${\;w}_{sf}$ is the front road input and $\;w_{sr}$ is the rear road input.

The total damping energy (D) is the combined dissipation in the suspension elements linking the vehicle body to both wheels, as in equation (5).


$$\begin{array}{c} D=\frac{\text{1}}{\text{2}}c_{sf}({\dot{x}}_{s}+L_{f}\dot{\theta }-{\dot{x}}_{uf}{)}^{\text{2}}+\frac{\text{1}}{\text{2}}c_{sr}({\dot{x}}_{s}-L_{r}\dot{\theta }-{\dot{x}}_{ur}{)}^{\text{2}} \end{array}$$ (5)

Where D is the total dissipation energy, $c_{sf}\;is\;the\;$front suspension damping coefficient and $c_{sr}\;is\;the\;$rear suspension damping coefficient.

#### 2.1.1. Mathematical modeling of ASS.

Dynamic modeling for ASS has an external actuator force inserted parallel to the passive elements. So, to compute the equations of motion for ASS, apply the Lagrangian equation. Then, the following equations (6)-(9) are developed.

1. For a vehicle body heave motion:

$$\begin{array}{c} m_{s}{\ddot{x}}_{s}=-(c_{sf}({\dot{x}}_{s}-{\dot{x}}_{uf}-L_{f}\dot{\theta })+k_{sf}(x_{s}-x_{uf}-L_{f}\theta )+c_{sr}({\dot{x}}_{s}-{\dot{x}}_{ur}+L_{r}\dot{\theta })+k_{sr}(x_{s}-x_{ur}+L_{r}\theta )-F_{sf}-F_{sr}) \end{array}$$ (6)

2. for a vehicle body pitching motion:

$$\begin{array}{c} J\ddot{\theta }=-L_{f}c_{sf}({\dot{x}}_{s}-{\dot{x}}_{uf}-L_{f}\dot{\theta })-L_{f}k_{sf}(x_{s}-x_{uf}-L_{f}\theta )+L_{r}c_{sr}({\dot{x}}_{s}-{\dot{x}}_{ur}+L_{r}\dot{\theta })+L_{r}k_{sr}(x_{s}-x_{ur}+L_{r}\theta )+L_{f}F_{sf}-L_{r}F_{sr} \end{array}$$ (7)

3. For the front wheel heave motion:

$$\begin{array}{c} m_{uf}{\ddot{x}}_{uf}=(c_{sf}({\dot{x}}_{s}-{\dot{x}}_{uf}+L_{f}\dot{\theta })+k_{sf}(x_{s}-x_{uf}+L_{f}\theta )-k_{uf}(x_{uf}-w_{sf})-F_{sf} \end{array}$$ (8)

4. For the rear wheel heave motion:

$$\begin{array}{c} m_{ur}{\ddot{x}}_{ur}=c_{sr}({\dot{x}}_{s}-{\dot{x}}_{ur}+L_{r}\dot{\theta })+k_{sr}(x_{s}-x_{ur}+L_{r}\theta )-k_{ur}(x_{ur}-w_{sr})-F_{sr} \end{array}$$ (9)

#### 2.1.2. State space model for half-car ASS.

The model is expressed in state-space form, as shown in equation (10).


$$\begin{array}{c} \dot{X}=AX+B_{u}u+B_{w}w \end{array}$$ (10)

Where X is the state vector, w is the control input (road disturbance), and u is the control input of the actuator forces, a continuous function represents the uncertainties due to the mismatch. All these equations can be expressed in matrix form as presented in equations (11)–(13).

**State Vector**

$$\begin{array}{c} X=[\begin{array}{c} x_{s}\\ \dot{x_{s}}\\ \theta \\ \dot{\theta }\\ x_{uf}\\ \dot{x_{uf}}\\ x_{ur}\\ \dot{x_{ur}} \end{array}]\;=[\begin{array}{c} x_{\text{1}}\\ x_{\text{2}}\\ x_{\text{3}}\\ x_{\text{4}}\\ x_{\text{5}}\\ x_{\text{6}}\\ x_{\text{7}}\\ x_{\text{8}} \end{array}] \end{array}$$ (11)

**System Matrix A**

$$\begin{array}{c} A=[\begin{array}{cccccccc} \text{0} & \text{1} & \text{0} & \text{0} & \text{0} & \text{0} & \text{0} & \text{0}\\ \frac{{-k}_{sf\;}{-k}_{sr}}{m_{s}} & \frac{{-c}_{sf}{-c}_{sr}}{m_{s}} & \frac{{-k}_{sf}L_{f}{-k}_{sr}L_{r}}{m_{s}} & \frac{{-c}_{sf}L_{f}{-c}_{sr}L_{r}}{m_{s}} & \frac{k_{sf}}{m_{s}} & \frac{c_{sf}}{m_{s}} & \frac{k_{sr}}{m_{s}} & \frac{c_{sr}}{m_{s}}\\ \text{0} & \text{0} & \text{0} & \text{1} & \text{0} & \text{0} & \text{0} & \text{0}\\ \frac{{-k}_{sf}L_{f}{-k}_{sr}L_{r}}{J} & \frac{{-c}_{sf}L_{f}{-c}_{sr}L_{r}}{J} & \frac{{-k}_{sf}{L^{\text{2}}}_{f}{-k}_{sr}{L^{\text{2}}}_{r}}{J} & \frac{{-c}_{sf}{L^{\text{2}}}_{f}{-c}_{sr}{L^{\text{2}}}_{r}}{J} & \frac{k_{sf}L_{f}}{J} & \frac{c_{sf}L_{f}}{J} & \frac{k_{sr}L_{r}}{J} & \frac{c_{sr}L_{r}}{J}\\ \text{0} & \text{0} & \text{0} & \text{0} & \text{0} & \text{1} & \text{0} & \text{0}\\ \frac{k_{sf}}{m_{uf}} & \frac{c_{sf}}{m_{uf}} & \frac{k_{sf}L_{f}}{m_{uf}} & \frac{c_{sf}L_{f}}{m_{uf}} & \frac{{-k}_{uf}{-k}_{sf}}{m_{uf}} & \frac{{-c}_{sf}}{m_{uf}} & \text{0} & \text{0}\\ \text{0} & \text{0} & \text{0} & \text{0} & \text{0} & \text{0} & \text{0} & \text{1}\\ \frac{k_{sr}}{m_{ur}} & \frac{c_{sr}}{m_{ur}} & \frac{{-k}_{sr}L_{r}}{m_{ur}} & \frac{{-c}_{sr}L_{r}}{m_{ur}} & \text{0} & \text{0} & \frac{{-k}_{ur}{-k}_{sr}}{m_{ur}} & \frac{{-c}_{sr}}{m_{ur}} \end{array}] \end{array}$$ (12)

$$\begin{array}{c} \;W=[\begin{array}{c} w_{sf}\\ w_{sr} \end{array}]{\;\;\;B}_{w}=[\begin{array}{cc} \text{0} & \text{0}\\ \text{0} & \text{0}\\ \text{0} & \text{0}\\ \text{0} & \text{0}\\ \text{0} & \text{0}\\ \frac{k_{uf}}{m_{uf}} & \text{0}\\ \text{0} & \text{0}\\ \text{0} & \frac{k_{ur}}{m_{ur}} \end{array}],B_{u}=[\begin{array}{cc} \text{0} & \text{0}\\ \frac{\text{1}}{m_{s}} & \frac{\text{1}}{m_{s}}\\ \text{0} & \text{0}\\ \frac{L_{f}}{J} & \frac{{-L}_{r}}{J}\\ \text{0} & \text{0}\\ \frac{-\text{1}}{m_{uf}} & \text{0}\\ \text{0} & \text{0}\\ \text{0} & \frac{-\text{1}}{m_{ur}} \end{array}]\;\;\;u=[\begin{array}{c} F_{sf}\\ F_{sr} \end{array}] \end{array}$$ (13)

### 2.2. ANFIS controller

The half-car suspension model controller is designed using ANFIS to improve ride comfort and ride handling. Unlike the quarter car model, which only captures the vertical dynamics of the vehicle, the half car model includes the front and rear dynamics of the vehicle. This includes the pitch dynamics to make the model more realistic. The goal of control is to restrict the displacement, acceleration, and deflection of the body. ANFIS effectively handles the suspension system's nonlinear and uncertain behavior by combining neural network learning and fuzzy logic reasoning.

For the calculation, we make the following two assumptions:

(a) The ANFIS model has two inputs and one output that are x, y, and f, respectively.
(b) It has only two rules (R_1_ and R_2_).

The ANFIS controller rules are based on the Tagaki-Sugeno model as follows:


$$\begin{array}{c} R_{\text{1}}:\text{\textasciitilde{}If\textasciitilde{}}x\;is\text{\textasciitilde{}}A_{\text{1}}\text{\textasciitilde{}and\textasciitilde{}}y\text{\textasciitilde{}is\textasciitilde{}}B_{\text{1}},\text{\textasciitilde{}then\textasciitilde{}}f_{\text{1}}=p_{\text{1}}x+q_{\text{1}}y+r_{\text{1}} \end{array}$$ (14)

$$\begin{array}{c} R_{2}:\text{\textasciitilde{}If\textasciitilde{}}x\;is\text{\textasciitilde{}}A_{2}\text{\textasciitilde{}and\textasciitilde{}}y\text{\textasciitilde{}is\textasciitilde{}}B_{2},\text{\textasciitilde{}then\textasciitilde{}}f_{2}=p_{2}x+q_{2}y+r_{2} \end{array}$$ (15)

Where x and y are input variables, Ai and Bi are the MFs of each input x and y, f is the output (linguistic variable), pi, qi, ri are linear parameters of the Tagaki-Sugeno Fuzzy model.

ANFIS Architecture is a five-layer architecture with the first layer as an input, and the last layer as an output. The network’s first and final layers serve as input and output nodes, while the hidden layers comprise membership functions. Square (adaptive) nodes contain tunable parameters, whereas circular (fixed) nodes have constant parameters [26]. The function of each layer is described as follows:

Layer 1 (Input/Fuzzification layer): This layer consists of square nodes that receive input values and convert them into fuzzy sets by computing their corresponding membership degrees. It maps crisp inputs into fuzzy representations using membership functions associated with linguistic terms such as “small” or “large”.


$$\begin{array}{c} {O_{i}}^{\text{1}}=\mu A_{i}\left(x\right),i=1,2\;\;\;\;\;{O_{i}}^{\text{1}}=\mu B_{i}\left(y\right),i=1,2 \end{array}$$ (16)

Where x donates the input to node i, while A_i_ represents the corresponding linguistic term (such as small, large, etc.) associated with that node function. In this context, A_i_ is defined through a membership function, which quantifies the degree to which a given input x belongs to or satisfies the fuzzy set A_i_.

Layer 2 (Rule layer): this layer contains fixed nodes labeled Π (product), which multiply incoming signals to determine the firing strength of each fuzzy rule. The output of each node represents the activation level of the corresponding rule.


$$\begin{array}{c} {O_{i}}^{\text{2}}=w_{i}=\mu A_{i}\left(x\right)*\mu B_{i}\left(y\right),i=1,2 \end{array}$$ (17)

Layer 3 (Normalization layer): this layer is represented by N (Norm), which computes the normalized firing strengths by dividing each rule’s firing strength by the total sum of all firing strengths, as presented in equation 18.


$$\begin{array}{c} {O_{i}}^{\text{3}}=\overset{―}{w_{i}}=\frac{w_{i}}{w_{\text{1}}+w_{\text{2}}} \end{array}$$ (18)

Layer 4 (Consequent/Defuzzification layer): each node contains a linear function that processes the normalized inputs to produce rule outputs as a linear combination of input variables plus a constant term. These outputs represent defuzzified values and are passed to the final layer.


$$\begin{array}{c} {O_{i}}^{\text{4}}=\overset{―}{w_{i}}fi=\overset{―}{w_{i}}(p_{i}x+q_{i}y+r_{i}) \end{array}$$ (19)

Layer 5 (output layer): this summation layer aggregates all rule outputs to generate the final ANFIS output, as presented in equation 20.


$$\begin{array}{c} {O_{i}}^{\text{5}}=\sum \overset{―}{w_{i}}f_{i}=\sum \overset{―}{w_{i}}(p_{i}x+q_{i}y+r_{i}) \end{array}$$ (20)

The adaptable parameters of ANFIS {$a_{i}{,b}_{i},c_{i},p_{i}{,q}_{i}{,r}_{i}$} are optimized by minimizing the performance index based on the training dataset P as given in equation 21.


$$\begin{array}{c} E=\sum_{p=1}^{p}E_{p} \end{array}$$ (21)

Where P is the entire training dataset and, $E_{p}$ represents the error between the desired and the predicted outputs for the$\;p^{th}$ sample, as given in equation 22.


$$\begin{array}{c} E_{p}=T_{p}-f_{p} \end{array}$$ (22)

Where $T_{p}$ the $p^{th}$ desired output and $f_{p}$ the $p^{th}$actual output of the ANFIS model.

Parameter tuning is performed using the steepest descent method for premise parameters $\{a_{i}{,b}_{i},c_{i}$}, while consequent parameters are estimated using the least squares method {$a_{i}{,b}_{i},c_{i},p_{i}{,q}_{i}{,r}_{i}$} [27].

An ANFIS controller requires data to train the system. This data is obtained from the PID controller. The PID controller includes the road inputs used, the vehicle speed used, the method used to generate the training dataset, and the amount of training data generated for each scenario.

The control force, u (t), is generated as in equation 23.


$$\begin{array}{c} u\left(t\right)=k_{p}e\left(t\right)+k_{i}\int e\left(t\right).dt+k_{d}\frac{d}{dt}e\left(t\right) \end{array}$$ (23)

The PID control system output is acquired by tuning the$\;k_{p}$, $\;k_{i}$and $k_{d}$ parameters. The tracking error is computed as in equation 24.


$$\begin{array}{c} e\left(t\right)=r\left(t\right)-y\left(t\right) \end{array}$$ (24)

Where, e (t) = Tracking error, y (t) = Actual Output, r (t) = Desired Output

The control force is generated using a PID controller to achieve the desired system response. The controller gains, kp, ki, and kd, are optimized using a closed-loop tuning approach, where the suspension model is linearized to provide initial parameter estimates. Reducing peak overshoot by increasing response time leads to a trade-off with longer settling time; therefore, the gains are selected to balance transient performance and settling characteristics. For the given model, the optimized values are $k_{p}$=2000, $k_{i}$= 10, $k_{d}$ = 900 and $k_{p}$= 2000, $k_{i}$ = 10, $k_{d}$= 900 for front and rear suspension, respectively.

Using PID-generated data provides a practical teacher signal and makes the ANFIS training problem well posed, but it also creates a form of teacher bias. If the PID controller produces excessive overshoot, slow recovery, or inadequate actuator action for a particular road condition, those behaviors can be embedded in the training data. Consequently, the present ANFIS is better interpreted as a nonlinear approximation and refinement of the PID control policy than as a fully independent adaptive controller. Direct adaptive fuzzy-learning approaches can instead update controller parameters from tracking performance and uncertainty online; for example, adaptive fuzzy control for nonlinear systems has been formulated with explicit predefined-time and accuracy guarantees [27]. This alternative is promising for future work, but it was not implemented in the present study.

The ANFIS controller was trained using data generated from a PID-controlled half-car suspension system under different road conditions and vehicle speeds. The dataset includes error signals and corresponding optimal control forces, enabling the ANFIS controller to learn the nonlinear control behavior and improve suspension performance. In this study, 90% of the dataset is used for training and 10% for validation. The results indicate that the ANFIS model accurately predicts control force for unseen inputs. Five Gaussian input membership functions (MFs) were selected for training purposes for each input e and 𝑒̇, respectively, and a hybrid algorithm was used to train them. The shape of the selected membership function (MF) was as shown in Fig 2 for each input e and 𝑒̇.

**Fig 2:**
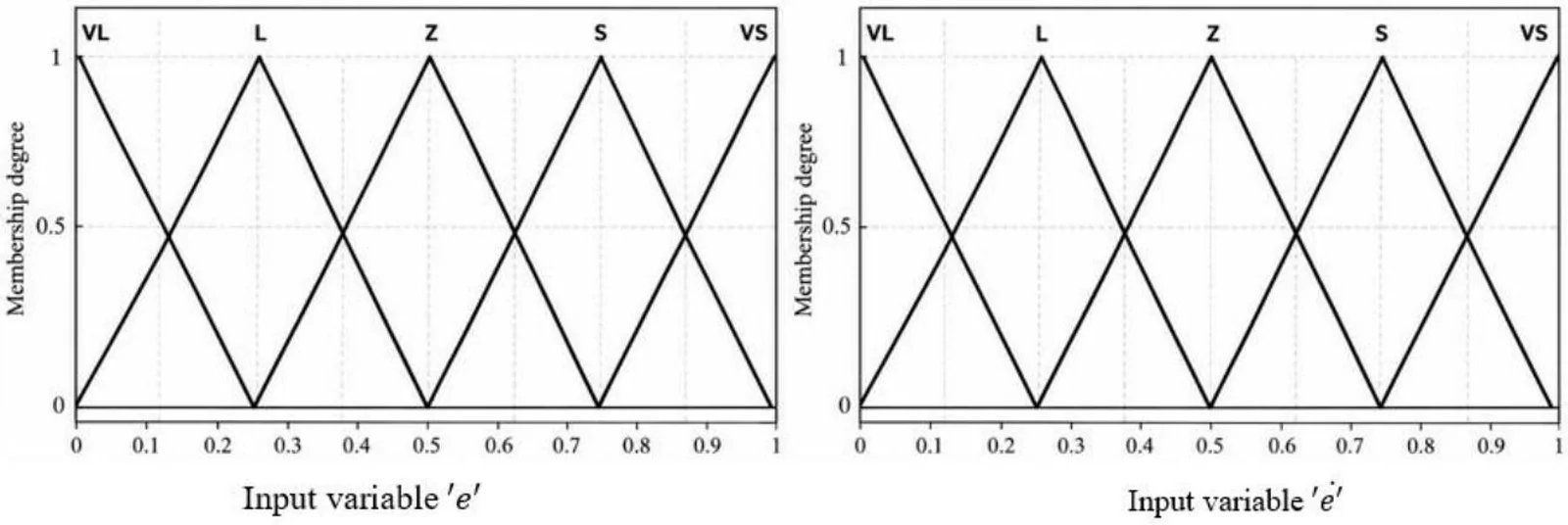
Membership functions for error signal (e) and derivative of error signal ($\dot{e})$.

The rule base consists of 25 rules formed by combining error (e) and change of error (Δe). The inputs and output variables are represented by five linguistic terms: NB, NS, ZE, PS, and PB. These rules determine the appropriate control force generated by the ASS actuator based on the suspension displacement error and its rate of change. Finally, we train the FIS file with hydride optimization, which assumes a zero-tolerance error.

For ANFIS hyperparameter selection, five Gaussian membership functions were retained for each of the two inputs because they provide five interpretable linguistic regions (NB, NS, ZE, PS, PB) and produce the 25-rule base used in the study. The choice of 150 epochs was based on the observed reduction of training error, which reached 0.000741 in the original training run. These values were not selected through a formal cross-validation or grid-search procedure. Accordingly, they should be regarded as design choices for the present dataset rather than globally optimal hyperparameters. A systematic sensitivity study varying the number/type of membership functions, epochs, training-validation split, and regularization would strengthen reproducibility and is recommended for future work.

Three columns and 1001 rows were used as mathematical data for training the ANFIS model, where the first and second columns were related to e and 𝑒̇, respectively, while the third column contained the slope magnitude values. For training data, the number of epochs was 150. The training error becomes 0.000741. The generated surface plot shows the relationship between two inputs and one output. These inputs are input one, which is the error, input two is the derivative of error, and the output is the control force (Fig 3). The final ANFIS architecture used in this study is shown in Fig 4.

**Fig 3:**
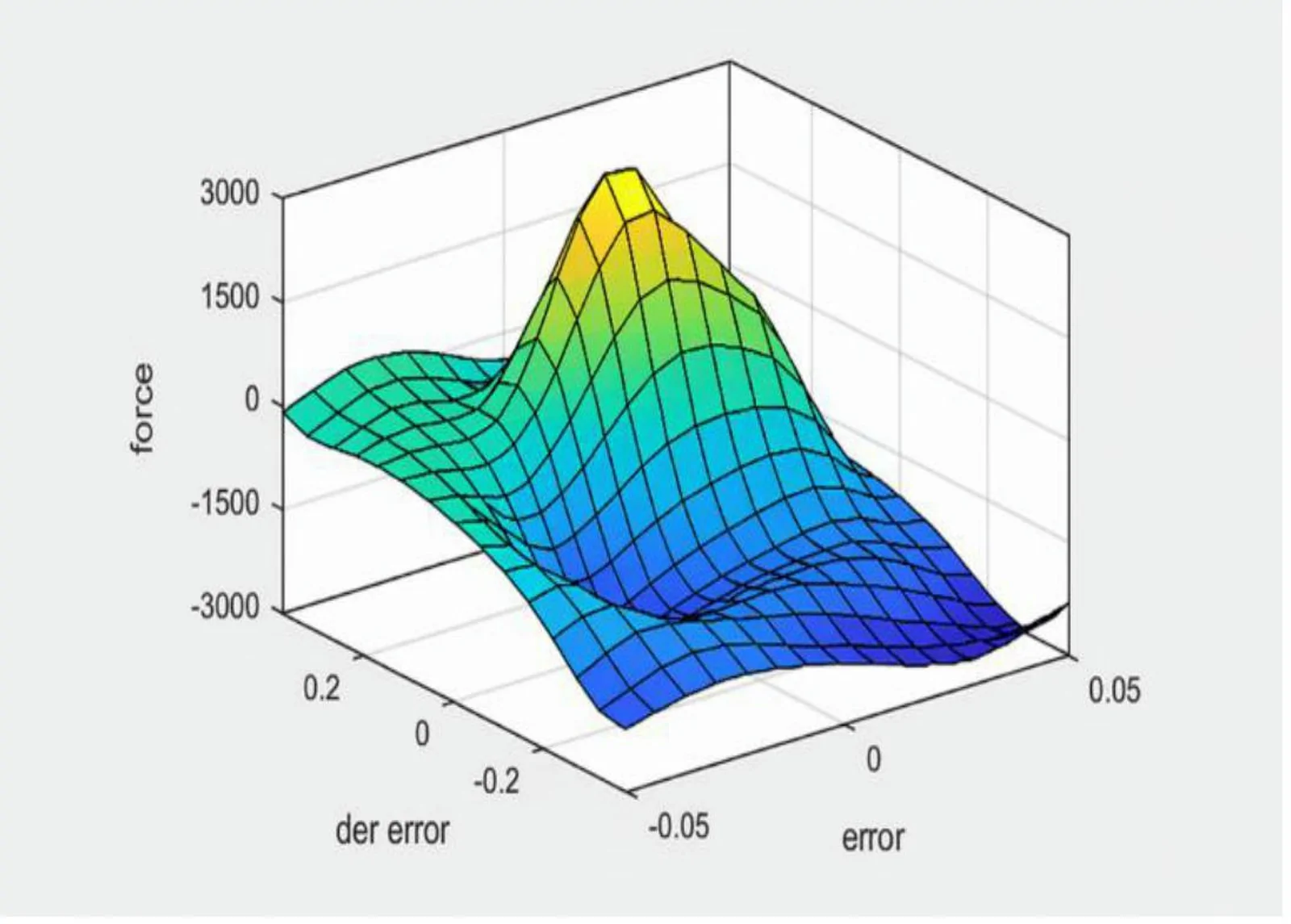
The generated surface plot.

**Fig 4:**
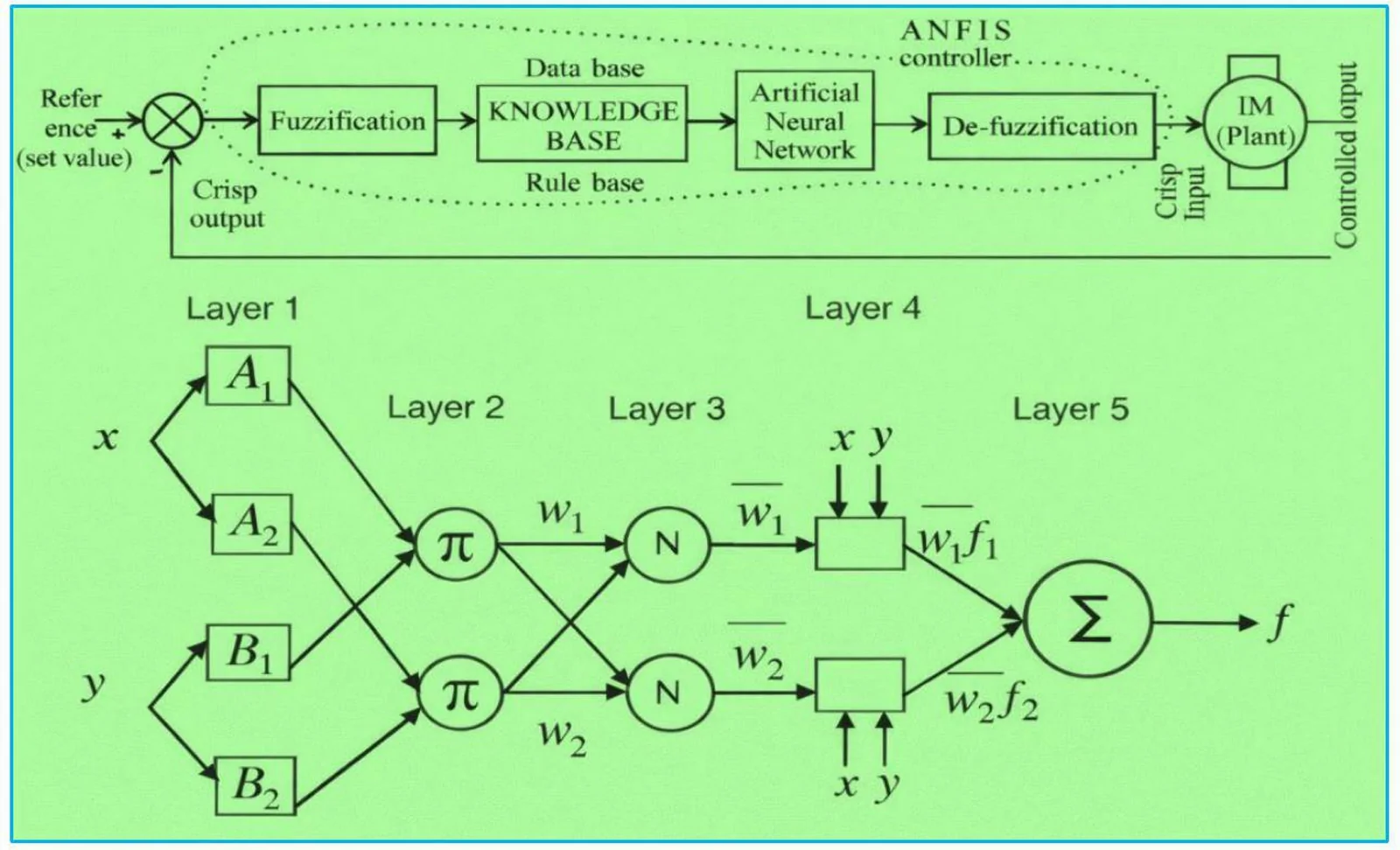
The final ANFIS Architecture [26].

The half-car ASS using an ANFIS controller, where the vehicle model (including sprung and unsprung masses) is subjected to road disturbances at the front and rear wheels, and system responses are fed back to compute the error and its derivative; these signals are processed by the ANFIS controller, which intelligently generates the optimal actuator forces applied to the suspension system to minimize vibrations, thereby improving ride comfort and maintaining vehicle stability by reducing body displacement, acceleration, and tire load variations.

#### 2.2.1. Double bump road profile.

The bump input road is designed to show the effect of vehicle speed limiters on driving comfort [28]. For this thesis, a sinusoidal road profile with two bumps was designed. The amplitude of the pump, denoted by A, is taken as 10 cm, and a sinusoidal shock with a frequency of 8 HZ is characterized by equations (25) and (26).


$$\begin{array}{c} w_{sf}=\left\{\begin{array}{c} @l@\left(A\left(1-cos8\pi t\right)\right)\;\text{1.0s}\leq \text{t}\leq \text{1.25s}\\ \left(A\left(1-cos8\pi t\right)\right)\;\text{4.0s}\leq \text{t}\leq \text{4.25s}\\ \;\;\;\;0\;\;\;\;\;\;\text{otherwise} \end{array}\right\} \end{array}$$ (25)

$$\begin{array}{c} w_{sr}=\left\{\begin{array}{c} @l@\left(A\left(1-cos8\pi t\right)\right)\;\text{2.0s}\leq \text{t}\leq 2\text{.25s}\\ \left(A\left(1-cos8\pi t\right)\right)\;\text{5.0s}\leq \text{t}\leq 5\text{.25s}\\ \;\;\;\;0\;\;\;\;\;\;\text{otherwise} \end{array}\right\} \end{array}$$ (26)

Equation (25) is an input disturbance for the front wheel, and Equation (26) is an input disturbance for the rear wheel (Fig 5).

**Fig 5:**
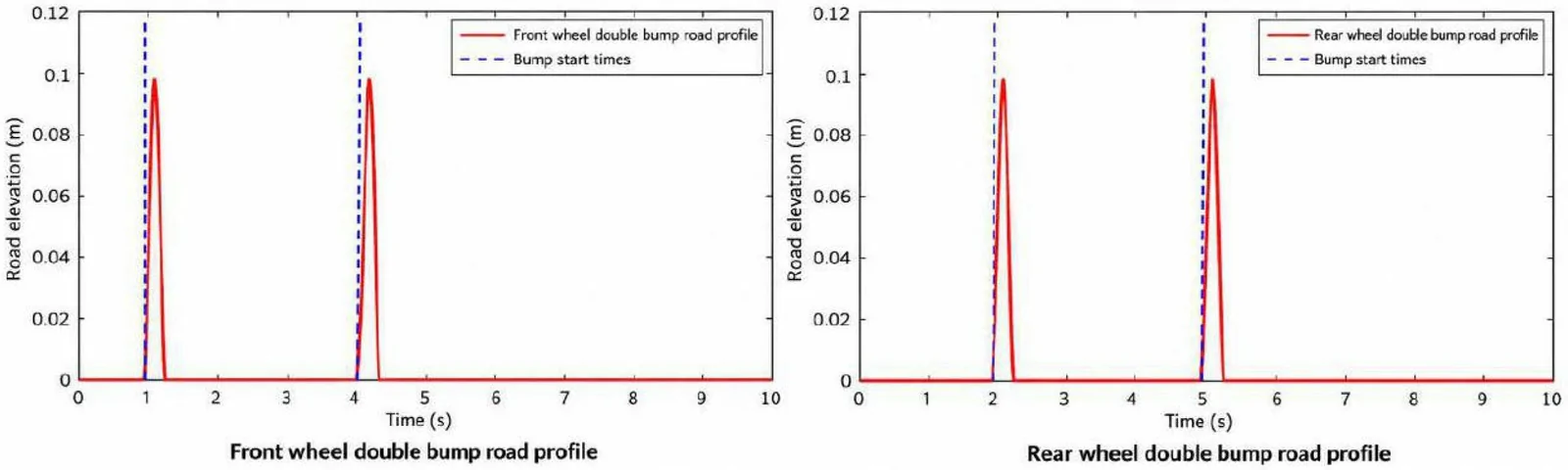
Front and rear-wheel bump road profile simulation.

#### 2.2.2. Random-type road profiles.

Based on ISO classifications of road roughness for this thesis, random type A (smooth) and random type C (rough) road inputs are selected at two operating vehicle speeds (20m/s and 10m/s) in order to show the effects of road roughness and vehicle speeds, besides ride comfort and road handling. This study described the integral approach for random white noise (noise power is 1, sample time is 0.1). The time delay between the front and rear wheels is determined based on the wheelbase length and the vehicle speed.


$$\begin{array}{c} t_{d}=\frac{L}{V} \end{array}$$ (27)

Where; $t_{d}$ is time delay, L is wheelbase length, and V is speed

The time delay is not constant for the front and rear; it changes with vehicle speed. Specifically, the delay is inversely proportional to speed, meaning that as the vehicle speed increases, the time delay decreases. At constant speed, the time-domain power spectrum behaves as white noise, and the road profile can be generated by integrating a white noise signal with a specified spectral density. The simulation is performed at 20m/s and 10m/s for both random road profiles. The simulations of class A and class C random road profiles are shown in Figs 6 and Fig 7, respectively. The result shows that a vehicle experiences high disturbances (high RMS value) when it runs at a higher speed than a lower speed, as well as when it runs on a rougher surface than a smooth surface road profile.

**Fig 6:**
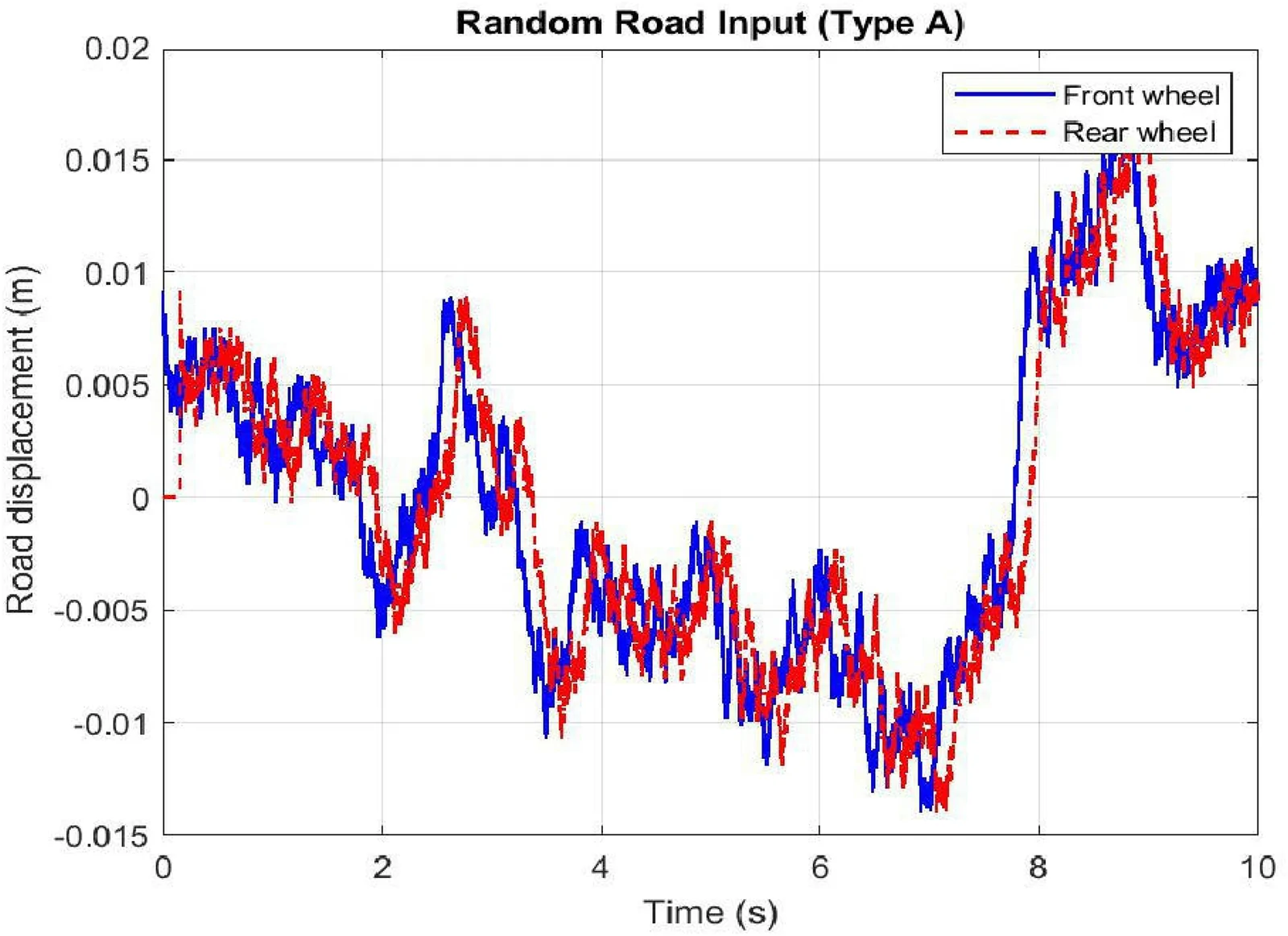
A Type random road profile simulation.

**Fig 7:**
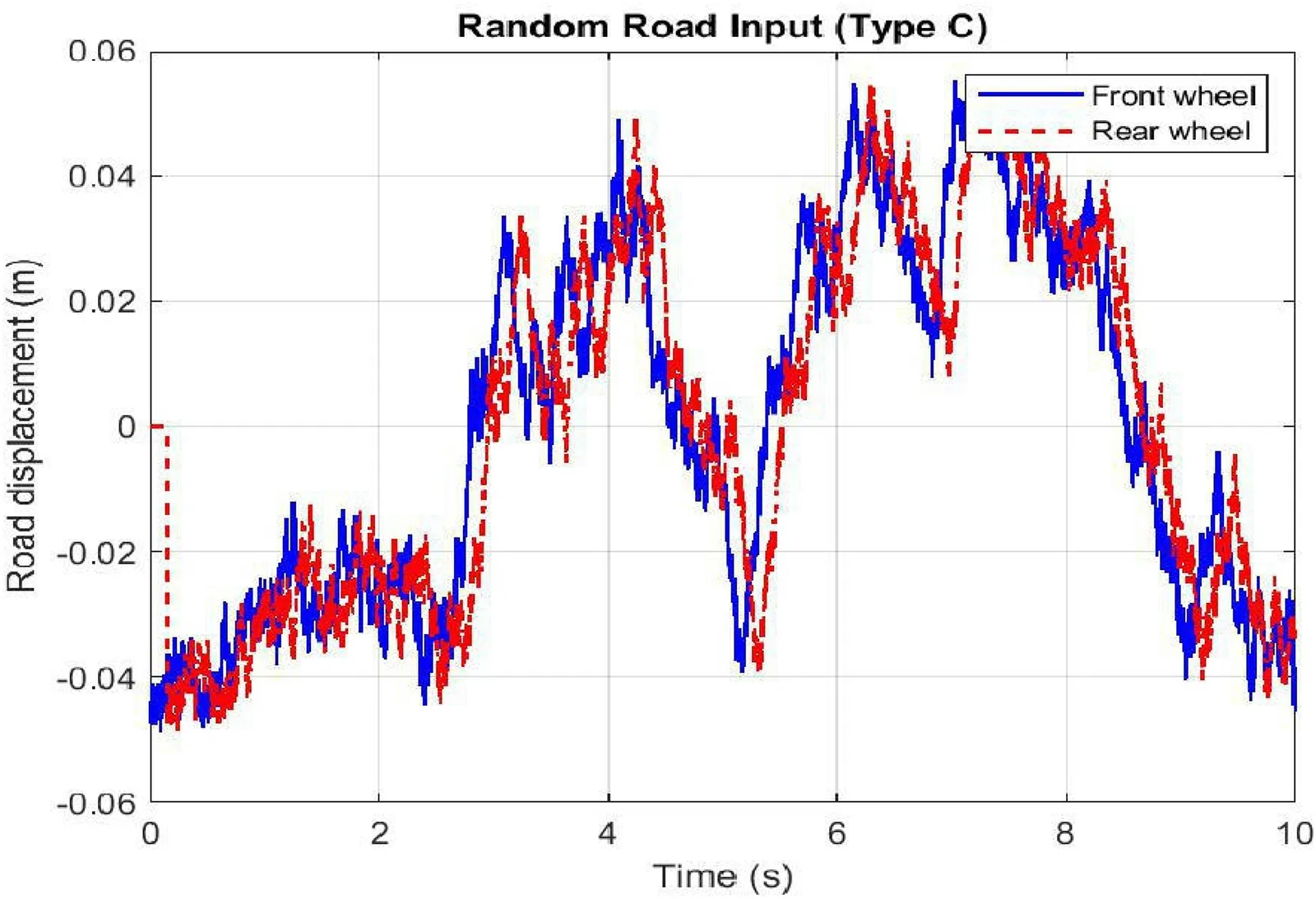
C Type random road profile simulations.

## 3. Results and discussion

The results are organized by road excitation and speed for PSS, PID-controlled ASS, and ANFIS-controlled ASS. Performance is assessed using body vertical displacement, body pitch displacement, vertical acceleration, front and rear suspension travel, pitch acceleration, and settling time. Percentage improvement is calculated as (baseline − controlled)/baseline × 100. Because each simulation condition represents a deterministic model realization rather than repeated experimental or stochastic trials, formal p-values and confidence intervals cannot be estimated from the available simulations without introducing unsupported assumptions. The reported percentages therefore describe deterministic effect sizes. For random-road cases, the RMS values summarize the simulated time histories; future Monte Carlo simulations with randomized road realizations and parameter uncertainty should be used to obtain confidence intervals and statistical significance estimates.

### 3.1. Double-bump sinusoidal road input simulation

This half-car model simulation for PSS and ASS with a PID and ANFIS controller based on double-bump sinusoidal road inputs at a vehicle speed at 10m/s. Figs 8–Fig 9, Fig 10, Fig 11, Fig 12, Fig 13 show the comparison of peak-to-peak displacement values of the PSS, ASS with PID controller, and ASS with ANFIS controller under double bump road excitation. The results in Tables 2 and 3 show that the ASS improves both ride comfort and vehicle handling stability by reducing the vibration amplitudes of the vehicle body and suspension components. The PSS exhibits the highest peak-to-peak displacement, indicating poor vibration isolation and larger body motion when the vehicle passes over road disturbances. In contrast, the PID-controlled active suspension reduces the displacement significantly by applying control forces through the actuators. However, the ANFIS-controlled suspension system achieves the lowest displacement values among all systems, demonstrating superior capability in suppressing vertical and pitch vibrations. This improvement is due to the adaptive learning and nonlinear control characteristics of the ANFIS controller, which enable the suspension system to respond more effectively to varying road excitations. Therefore, the ANFIS-based ASS provides the best performance in minimizing vehicle vibration, enhancing ride comfort, and improving vehicle stability compared with both the PSS and the PID-controlled ASS.

**Table 2:** Comparison of peak-to-peak displacement of PSS to ASS with PID and ANFIS controller simulations.

No	Parameter	PSS	ASS with PID	ASS with ANFIS	Improvement of PSS over PID (%)	Improvement of PSS over ANFIS (%)
**Ride comfort**						
1	Vehicle Vertical body displacement (m)	0.0435	0.0181	0.0084	58.4%	80.6%
2	Vehicle Rotational displacement (rad)	0.0492	0.035	0.023	28.8%	54%
3	Vehicle Vertical acceleration (m/s^2)	2.4	1.52	0.77	36.6%	67.9%
**Vehicle handling stability**						
4	Suspension travel at front wheel (m)	0.0652	0.016	0.009	75.5%	86.2%
5	Suspension travel at rear wheel (m)	0.0938	0.0181	0.0152	59.4%%	83.7%
6	RMS Pitch acceleration (rad/s^2)	5.90	3.80	1.95	35.5%	66.9%

**Table 3:** Comparison of settling time of PSS to ASS simulations.

No	Parameter	PSS (sec)	ASS with PID (sec)	ASS with ANFIS (sec)	Improvement of PID over PSS (%)	Improvement of ANFIS over PSS (%)
**Ride comfort**						
1	Vehicle Vertical body displacement	9	6.5	5.5	27.7%	38.8%
2	Vehicle Rotational body displacement	9.3	6.5	5.5	30.1%	40.8%
3	Vertical acceleration	9.08	7	6.5	22.9%	30.8%
**Vehicle handling stability**						
4	Suspension travel at the front wheel	8.76	5.78	5.5	33%	37.2%
5	Suspension travel at the rear wheel	8.28	6.48	5.5	21.7%	33.5%
6	Pitch acceleration	9.78	7.35	6.00	24.8%	38.6%

**Fig 8:**
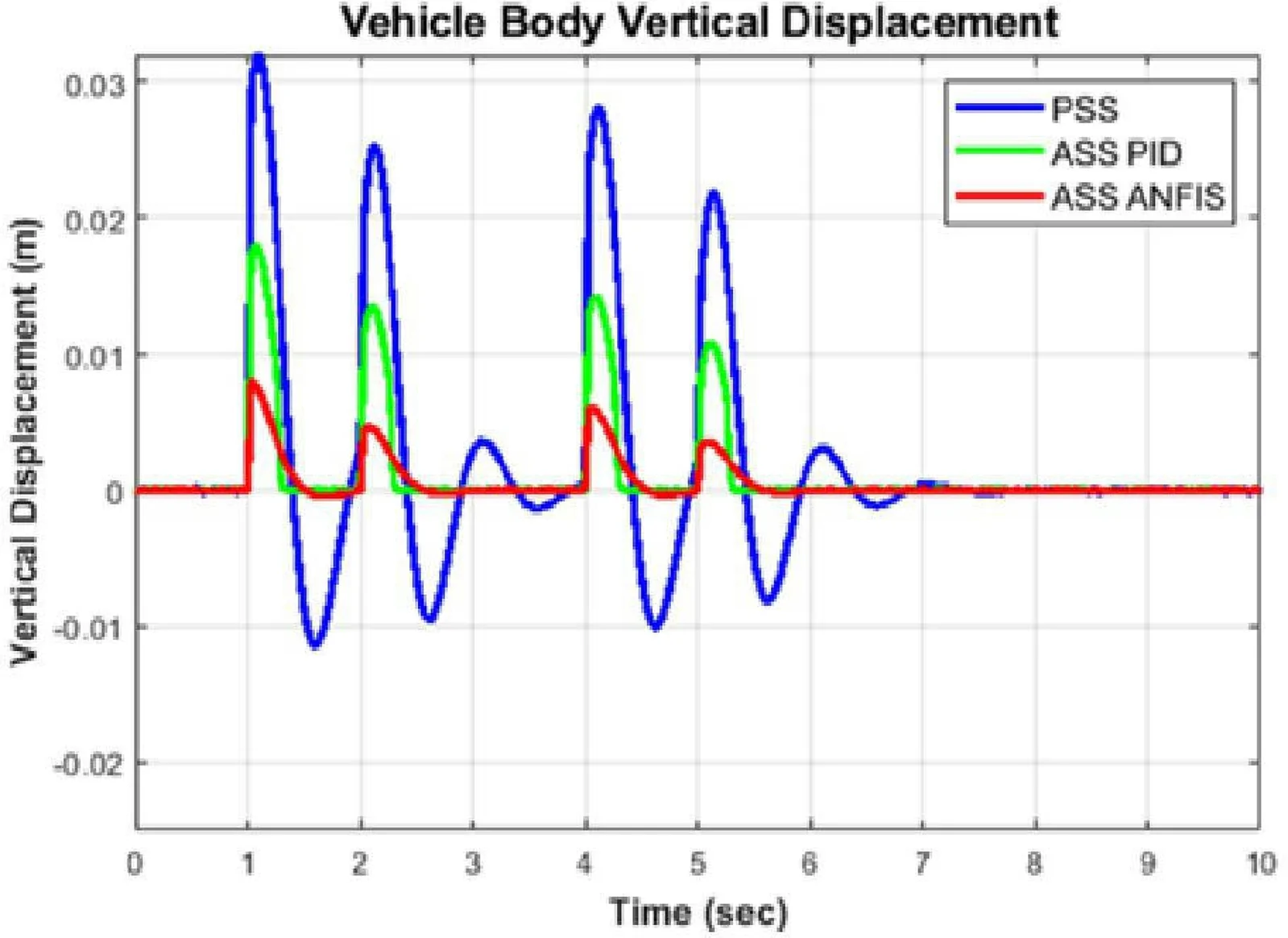
Vehicle body vertical displacement.

**Fig 9:**
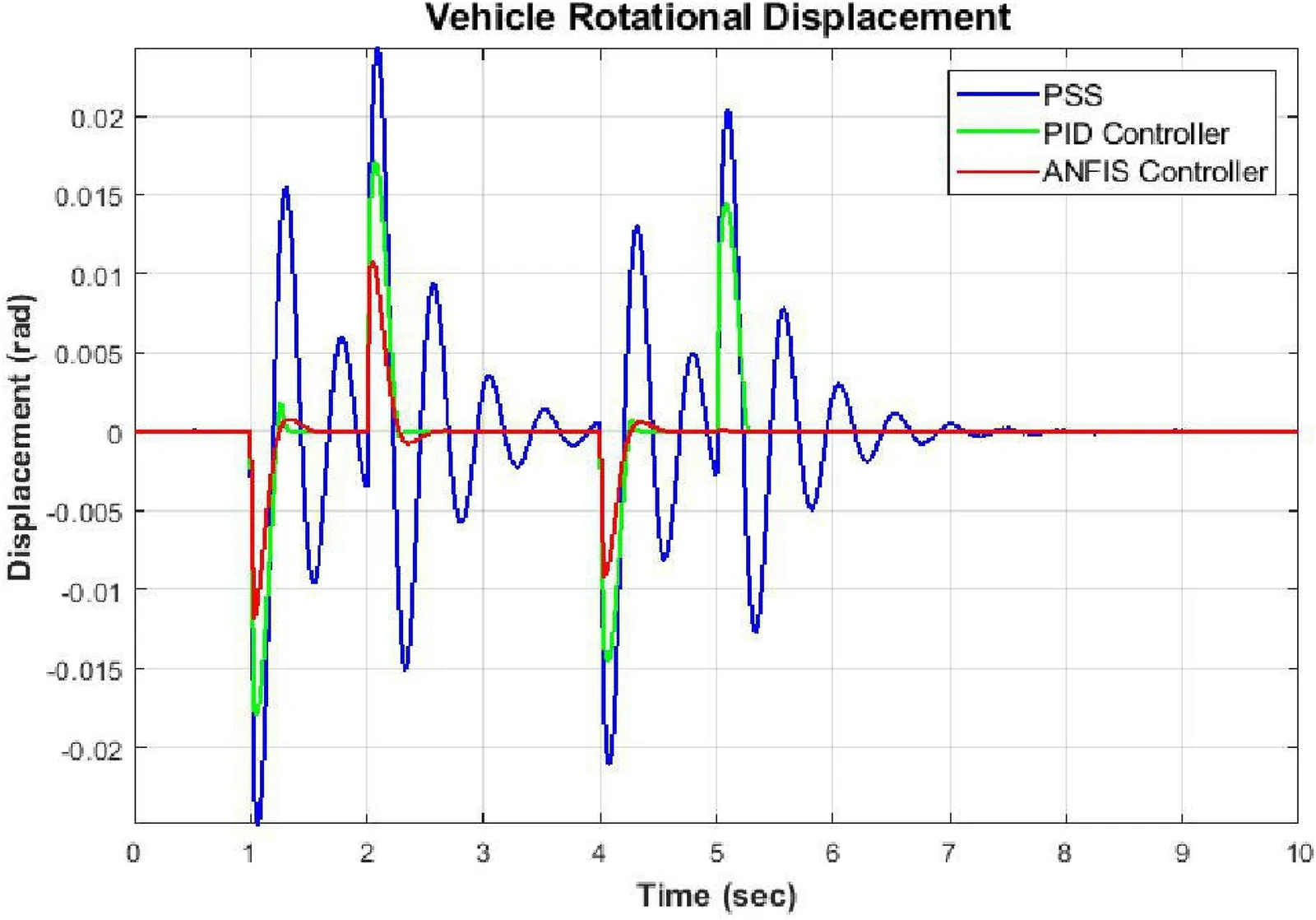
Vehicle rotational displacement.

**Fig 10:**
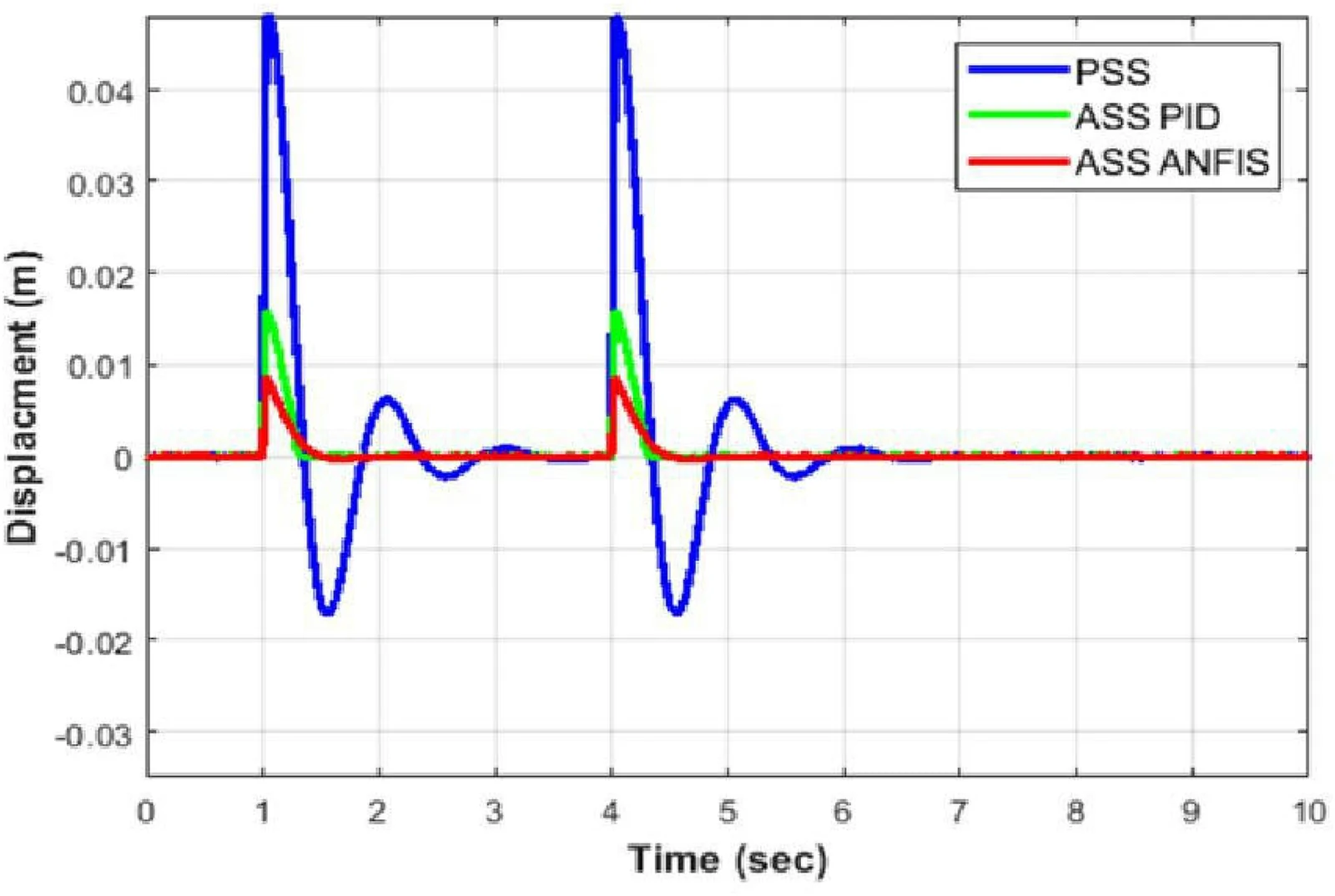
Front-wheel- Suspension travel.

**Fig 11:**
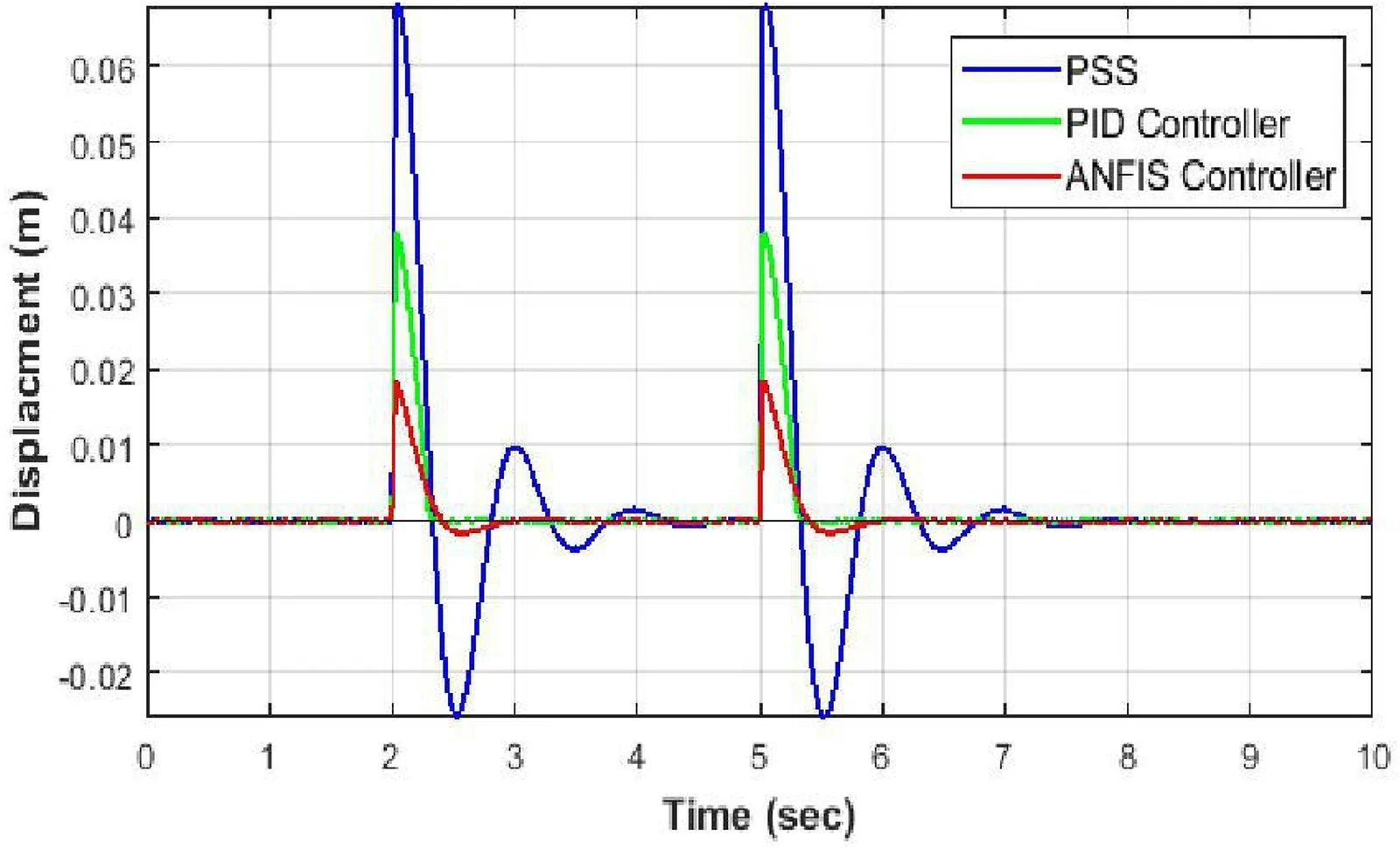
Rear-wheel Suspension travel.

**Fig 12:**
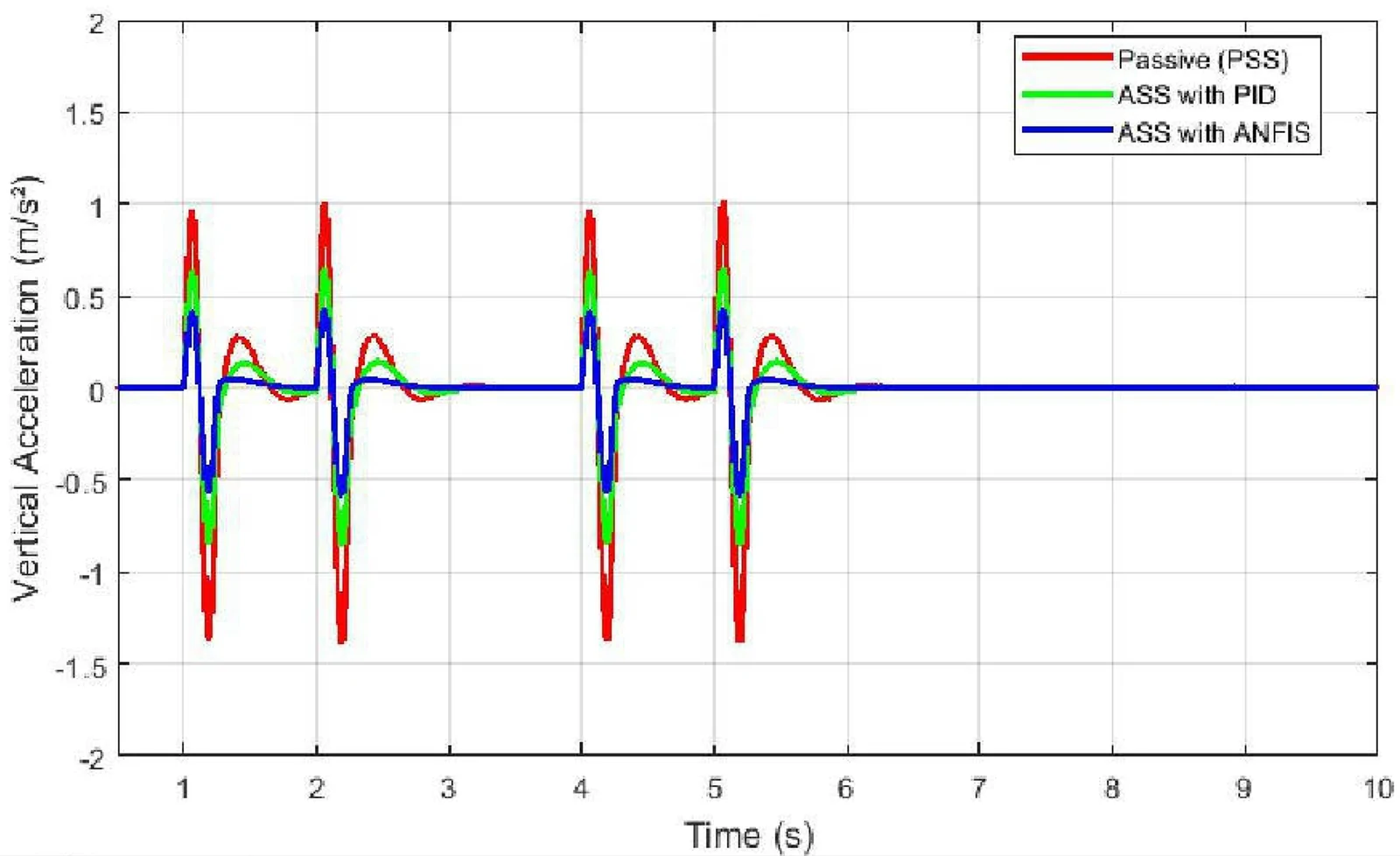
Vehicle body acceleration simulations.

**Fig 13:**
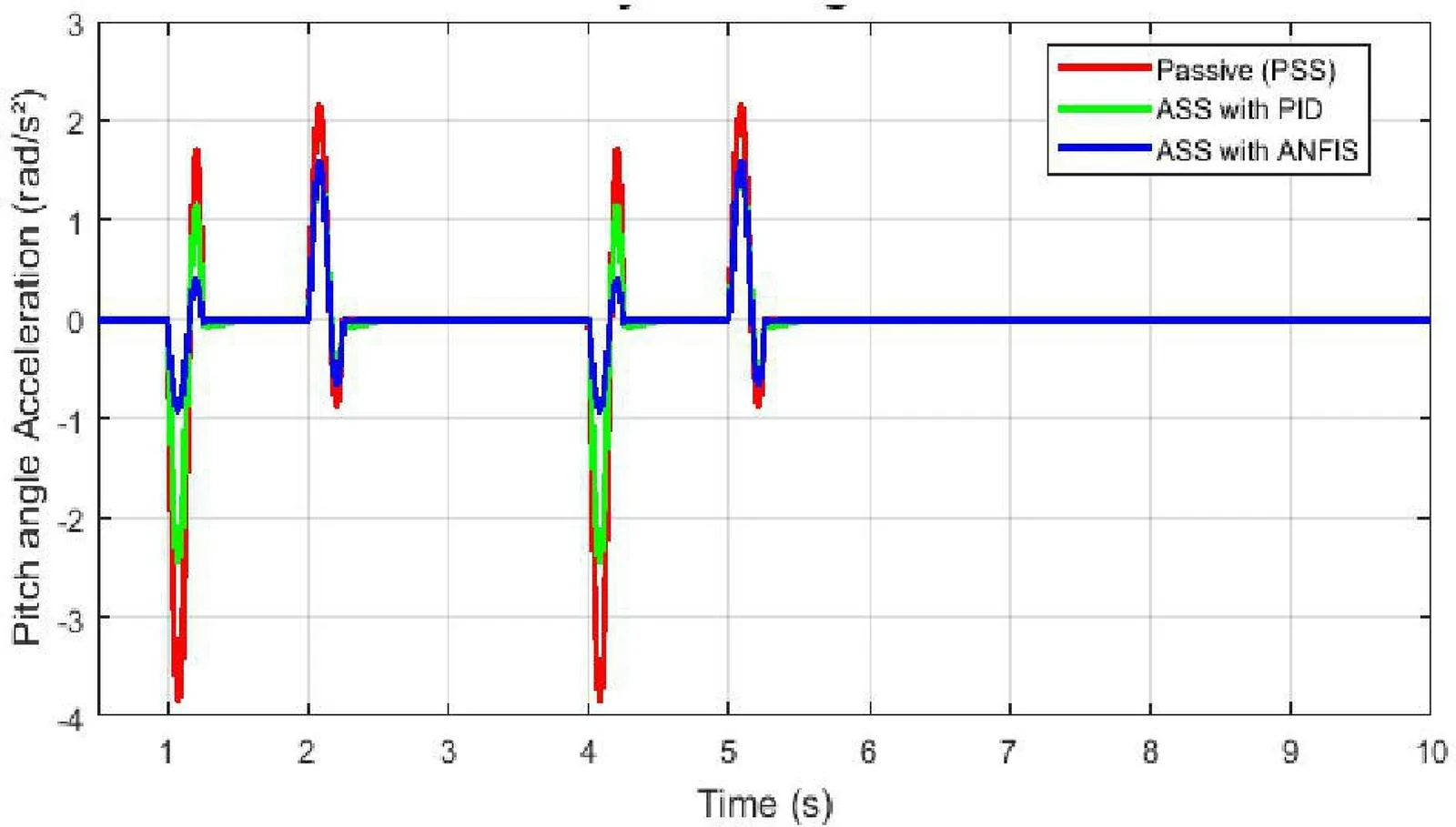
Vehicle body pitch angle acceleration.

The larger reduction in peak-to-peak displacement than in settling time is mechanically reasonable. Peak-to-peak displacement is dominated by the magnitude of the first few transient excursions immediately after the road disturbance, so the active actuator can strongly oppose the instantaneous body and wheel motion. Settling time, by contrast, is governed by the complete decay of the residual oscillatory energy of the coupled sprung and unsprung masses. Once the dominant transient has been reduced, the remaining response is still constrained by the natural frequencies and damping of the passive mechanical elements. Therefore, an approximately 80–86% reduction in displacement can coexist with a more modest 33–39% reduction in settling time. This distinction indicates that ANFIS is particularly effective at limiting excursion amplitude, while the underlying suspension dynamics continue to influence the time required for the response to decay.

### 3.2. Comparative simulation of PSS and ASS at a random Type A road profile with different vehicle speeds

The comparative simulation analysis of PSS and ASS at type A random road profiles based on 10 m/s and 20 m/s vehicle speeds has been performed to improve passengers’ ride comfort and vehicle handling stability. Figs 14–Fig 15, Fig 16, Fig 17 show the comparative performance of the PSS, ASS with PID controller, and ASS with ANFIS controller under ISO Type A random road profile at vehicle speeds of 10 m/s and 20 m/s. The results show that the ASS significantly improves ride comfort and vehicle handling stability compared to the PSS at both speeds. As shown in Table 4, at 10 m/s, the ANFIS controller achieved greater reductions in vehicle body displacement, rotational displacement, and suspension travel than the PID controller, demonstrating superior vibration suppression and stability performance. Similarly, at 20 m/s, the suspension responses increased due to higher road excitation caused by vehicle speed, but the ANFIS controller still maintained better control performance than PID by minimizing body motion and suspension deflection more effectively (Table 5). Increasing vehicle speed from 10 m/s to 20 m/s slightly reduces the effectiveness of all suspension systems because higher speed amplifies road-induced vibrations. However, the ANFIS-controlled active suspension consistently provides the best overall performance in terms of ride comfort and road holding, proving its capability to adapt to varying operating conditions and random road disturbances more efficiently than the conventional PID controller and PSS shown in Tables 4 and 5.

**Table 4:** Type A random road input improvement at 10 m/s.

No		Parameter	RMS of PSS	RMS of ASS with PID	RMS of ASS with ANFIS	Improvement of PID over PSS (%)	Improvement of ANFIS over PSS (%)
**Ride comfort**							
1	Vehicle Vertical body displacement (m)		0.028	0.013	0.006	53.5%	78.6%
2	Vehicle Rotational body displacement (rad)		0.0049	0.0023	0.0011	53.1%	77.5%
**Vehicle handling stability**							
3	Suspension travel at front wheel (m)		0.034	0.018	0.011	47.6%	67.6%
4	Suspension travel at rear wheel (m)		0.032	0.017	0.010	46.8%	68.7%

**Table 5:** Type A random road input improvement at 20 m/s.

No	Parameter	RMS of PSS	RMS of ASS with PID	RMS of ASS with ANFIS	Improvement of PID over PSS (%)	Improvement of ANFIS over PSS (%)
**Ride comfort**						
1	Vehicle Vertical body displacement (m)	0.040	0.020	0.010	50%	75%
2	Vehicle Rotational body displacement (rad)	0.0071	0.0035	0.0018	50.7%	74.6%
**Vehicle handling stability**						
3	Suspension travel at front wheel (m)	0.045	0.026	0.016	42.2%	64.4%
4	Suspension travel at rear wheel (m)	0.043	0.025	0.015	41.8%	65.1%

**Fig 14:**
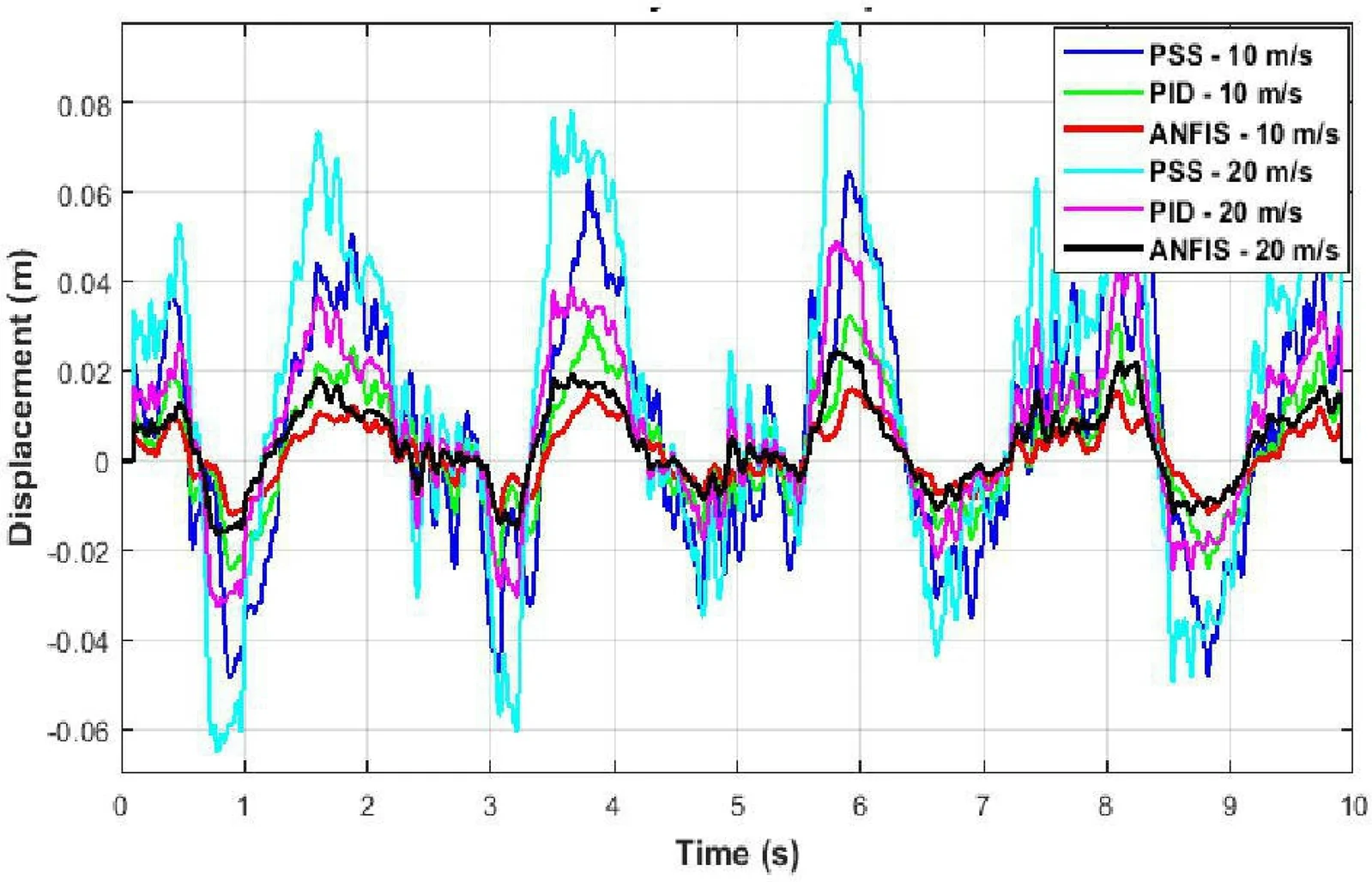
Vertical body heave displacement.

**Fig 15:**
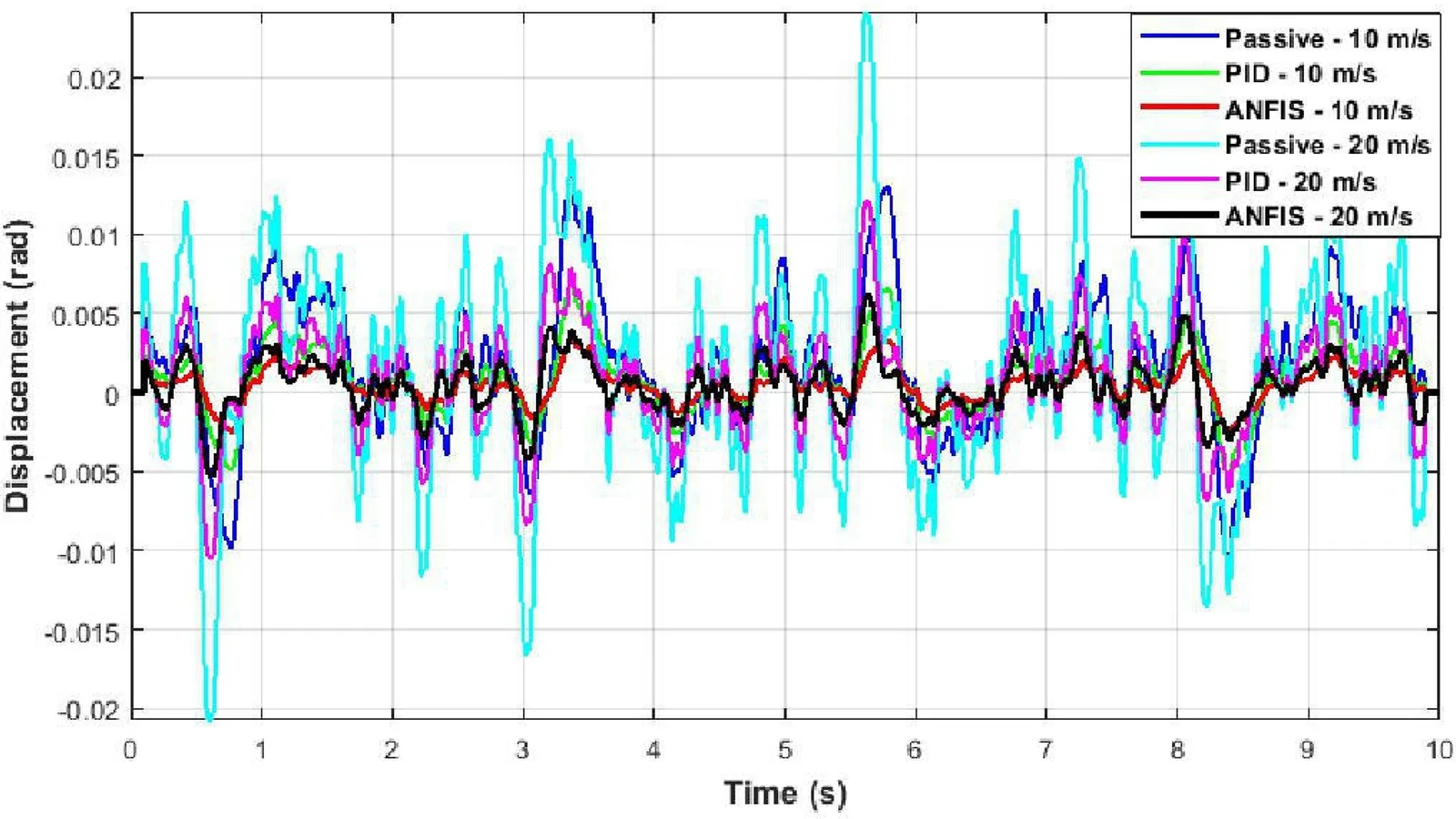
Vertical body pitch displacement.

**Fig 16:**
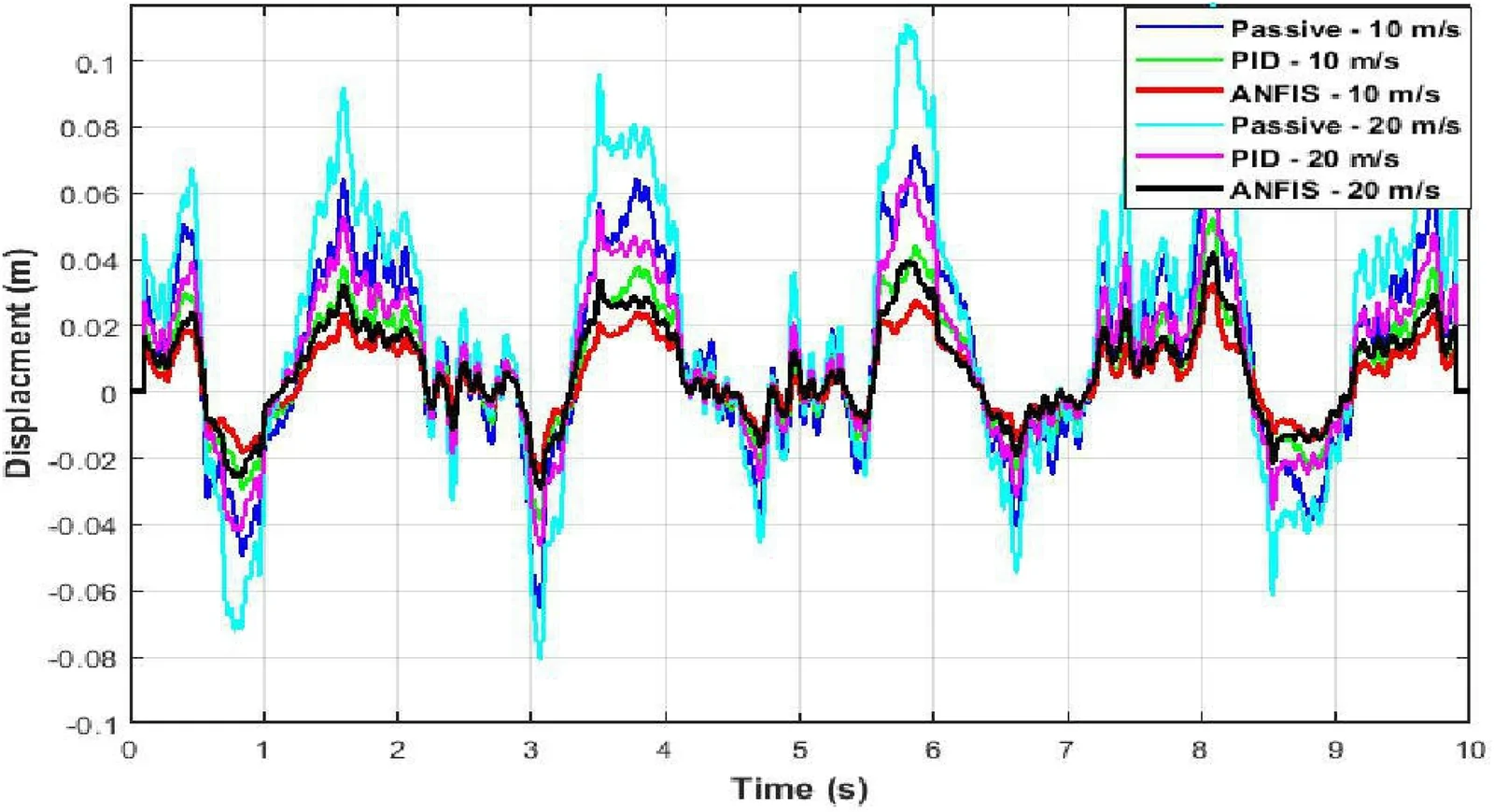
Suspension travel at front-wheel drive.

**Fig 17:**
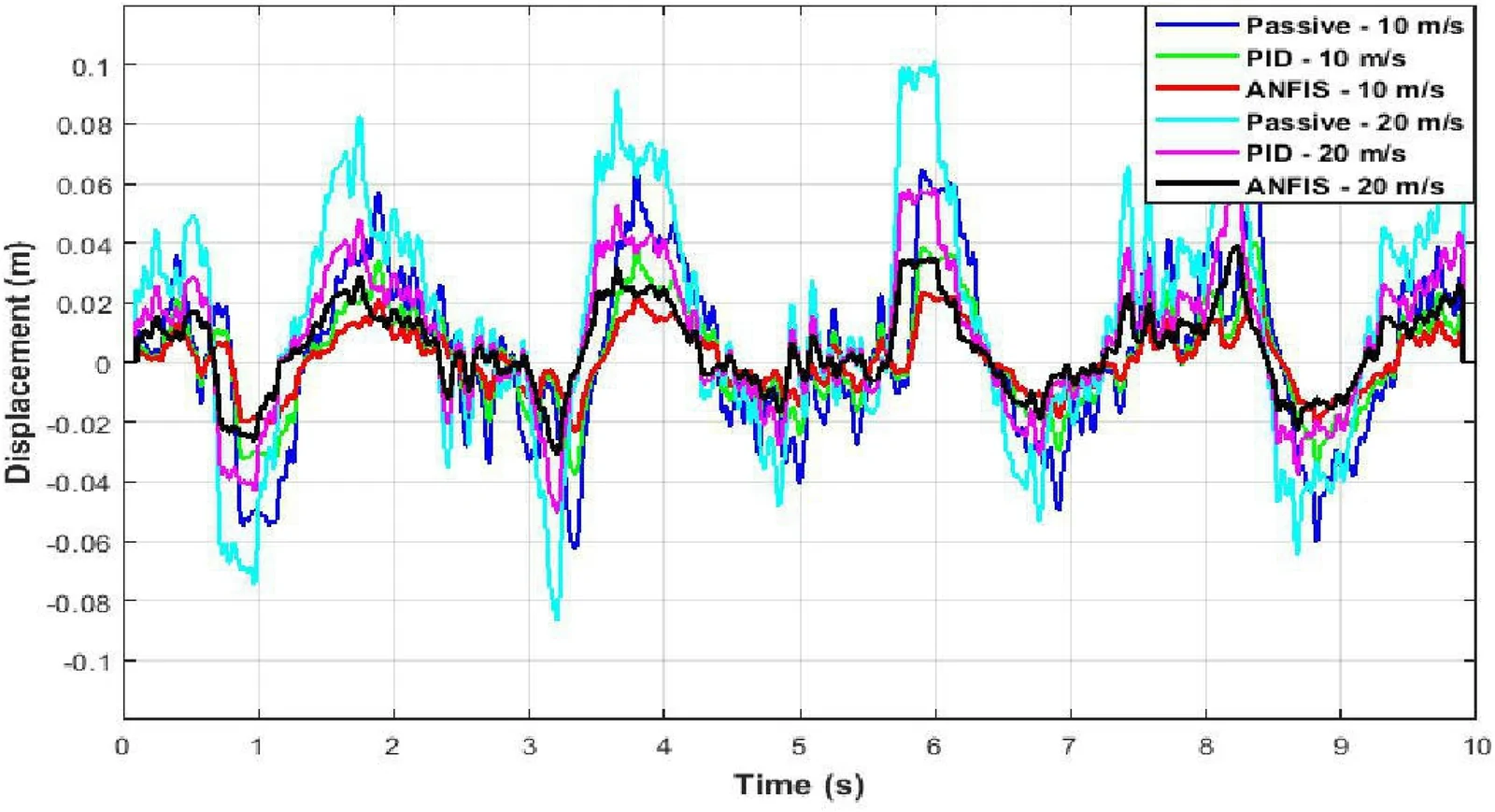
Suspension travel at the rear wheel drive.

Since the simulations result unweighted vertical acceleration and do not provide the complete frequency-weighted acceleration procedure, frequency weighting, exposure duration, or health/caution guidance required for a formal ISO 2631 assessment. The present results therefore demonstrate reduced vertical acceleration relative to PSS, but they should not be interpreted as formal ISO 2631 compliance. A future validation should calculate the appropriate frequency-weighted RMS acceleration and vibration dose measures for the relevant exposure period.

In addition to ride-comfort and handling performance, the control effort required by an active suspension system is an important consideration for practical implementation. Excessive actuator force and energy demand may offset the benefits obtained from vibration suppression. The determined RMS front and rear actuator-force demands ranged from 217–613 N and 424–720 N, respectively. The determined total average mechanical power increased from 47 W for the double-bump excitation at 10 m/s to 155 W for the Type C Road at 20 m/s. This increase is physically consistent with the greater suspension excitation associated with increased road roughness and vehicle speed. Using a standardized 10-s analysis interval, the estimated total actuator energy demand ranged from 470 to 1,550 J. Recent robust disturbance-rejection work emphasizes the treatment of uncertainty and measurement noise in control design [29], while integrated active-suspension studies explicitly include energy-saving objectives [17]. Therefore, this study results indicated that greater disturbance severity requires increased actuator effort even though the ANFIS controller maintains substantial reductions in body displacement, acceleration, and suspension travel.

### 3.3. Comparative simulation of PSS and ASS with PID and ANFIS controllers at a random Type C Road profile with different vehicle speeds

The comparative simulation analysis of PSS and ASS at type C random road profiles based on 10 m/s and 20 m/s vehicle speeds has been performed to improve passengers’ ride comfort and vehicle handling stability. Figs 18–Fig 19, Fig 20, Fig 21 show the PSS, ASS with PID controller, and ASS with ANFIS controller under an ISO Type C random road profile at vehicle speeds of 10 m/s and 20 m/s. Since Type C represents a rougher road condition than Type A, all suspension responses, such as vehicle body displacement, pitch motion, and suspension travel, show larger vibration amplitudes. However, both active suspension controllers significantly improve ride comfort and vehicle handling stability compared to the PSS. At 10 m/s, the ANFIS controller achieves greater reductions in vertical and rotational body motions as well as suspension travel than the PID controller, indicating better vibration isolation and road holding capability (Table 6). At 20 m/s, the vehicle experiences more severe dynamic excitation due to the combined effect of rough road conditions and higher speed, causing increased suspension responses in all systems (Table 7). Therefore, the ANFIS-controlled suspension maintains superior performance by effectively minimizing body vibrations and improving stability compared to the PID-controlled and passive systems. The effectiveness of the suspension system decreases slightly as vehicle speed increases, but the ANFIS controller consistently provides the best overall performance under rough road conditions, demonstrating its strong adaptability and robustness for improving ride comfort and handling stability, as shown in Tables 6 and 7.

**Table 6:** Type C random road input improvement at 10 m/s.

No	Parameter	RMS of PSS	RMS of ASS with PID	RMS of ASS with ANFIS	Improvement of PID over PSS	Improvement of ANFIS over PSS
**Ride comfort**						
1	Vehicle Vertical body displacement (m)	0.037	0.019	0.010	48.6%	72.6%
2	Vehicle Rotational body displacement (rad)	0.0074	0.0039	0.0020	47.3%	72.9%
**Vehicle handling stability**						
3	Suspension travel at front wheel (m)	0.034	0.020	0.012	41.2%	64.7%
4	Suspension travel at rear wheel (m)	0.031	0.018	0.011	41.9%	64.5%

**Table 7:** Type C random road input improvement at 20 m/s.

No	Parameter	RMS of PSS	RMS of ASS with PID	RMS of ASS with ANFIS	Improvement of PID over PSS (%)	Improvement of ANFIS over PSS (%)
**Ride comfort**						
1	Vehicle Vertical body displacement (m)	0.052	0.028	0.015	46.2%	71.2%
2	Vehicle Rotational body displacement (rad)	0.0106	0.0057	0.003	46.2%	71.7%
**Vehicle handling stability**						
3	Suspension travel at front wheel (m)	0.048	0.029	0.018	39.5%	62.5%
4	Suspension travel at rear wheel (m)	0.044	0.026	0.017	40.9%	61.3%

**Fig 18:**
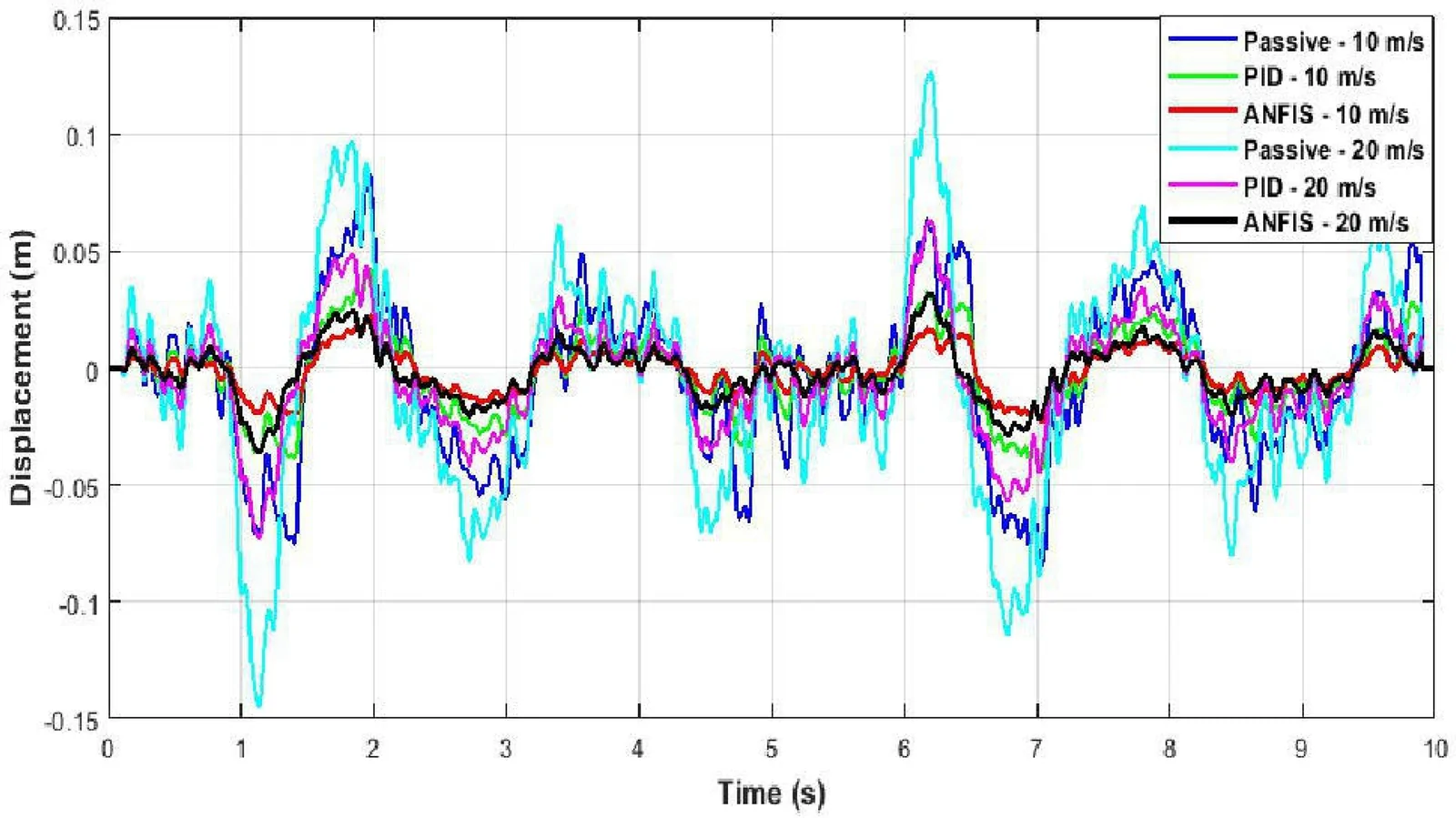
Vertical body heave displacement.

**Fig 19:**
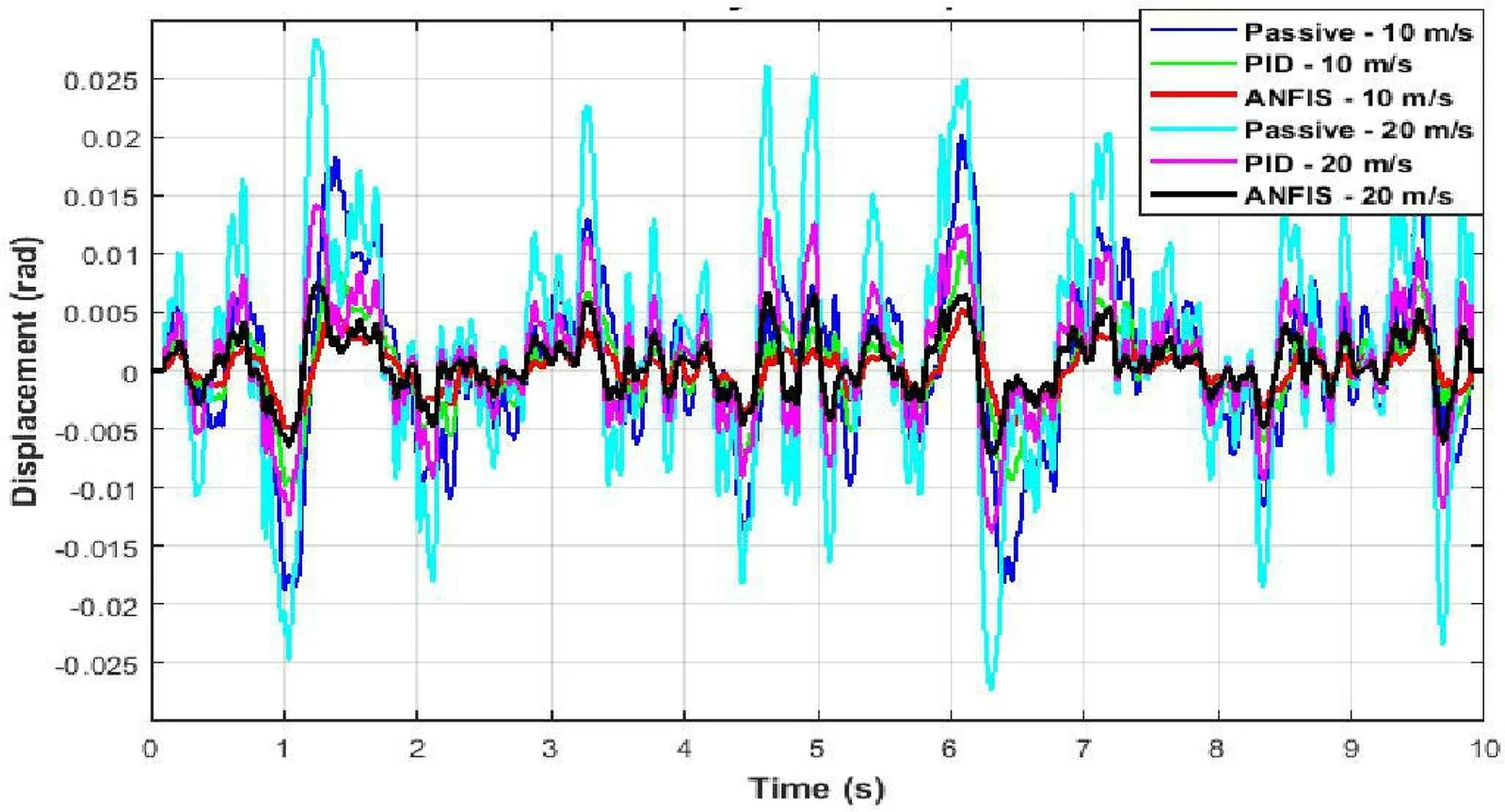
Vertical body pitch displacement.

**Fig 20:**
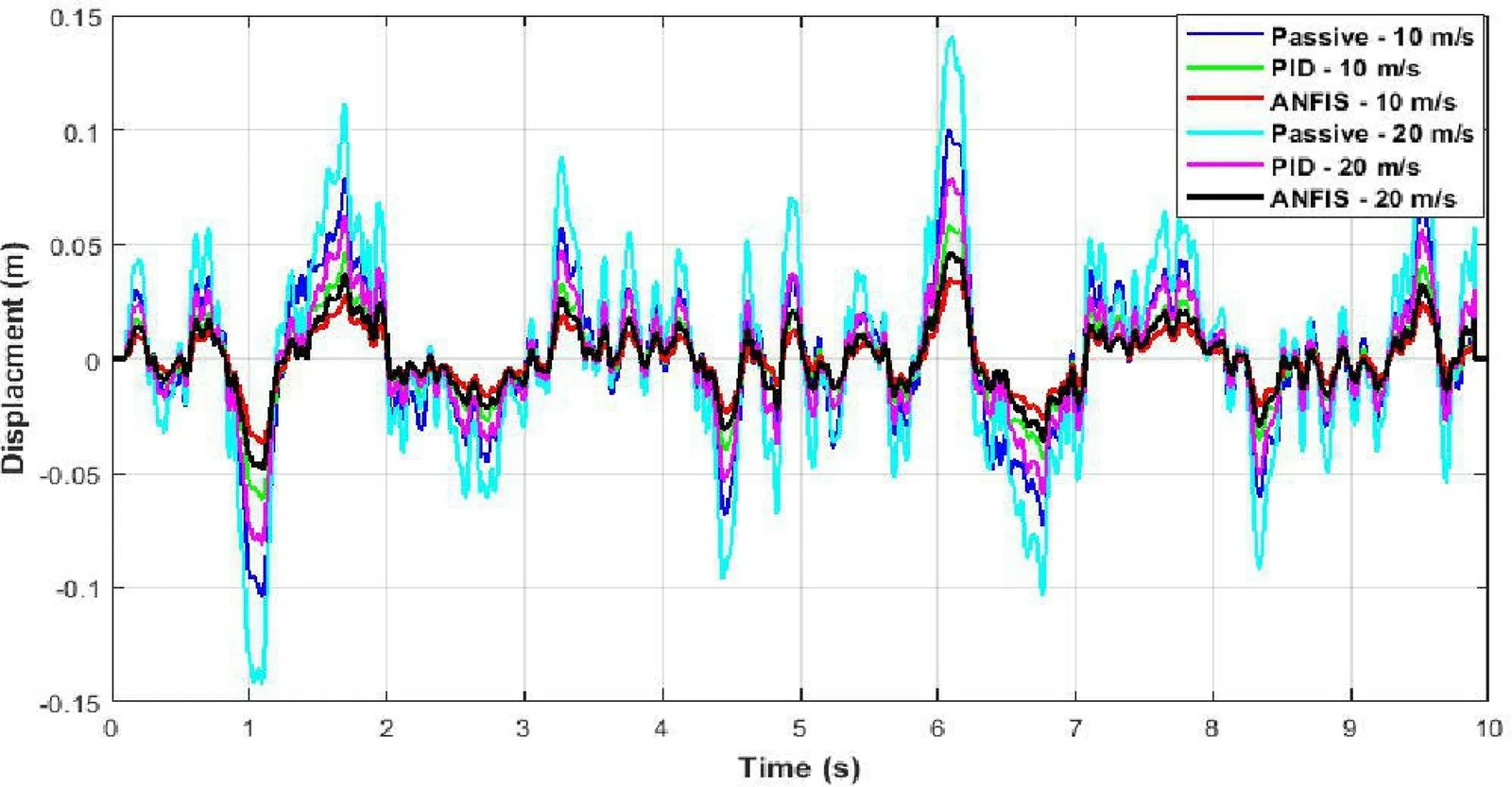
Suspension travel at front wheel drive.

**Fig 21:**
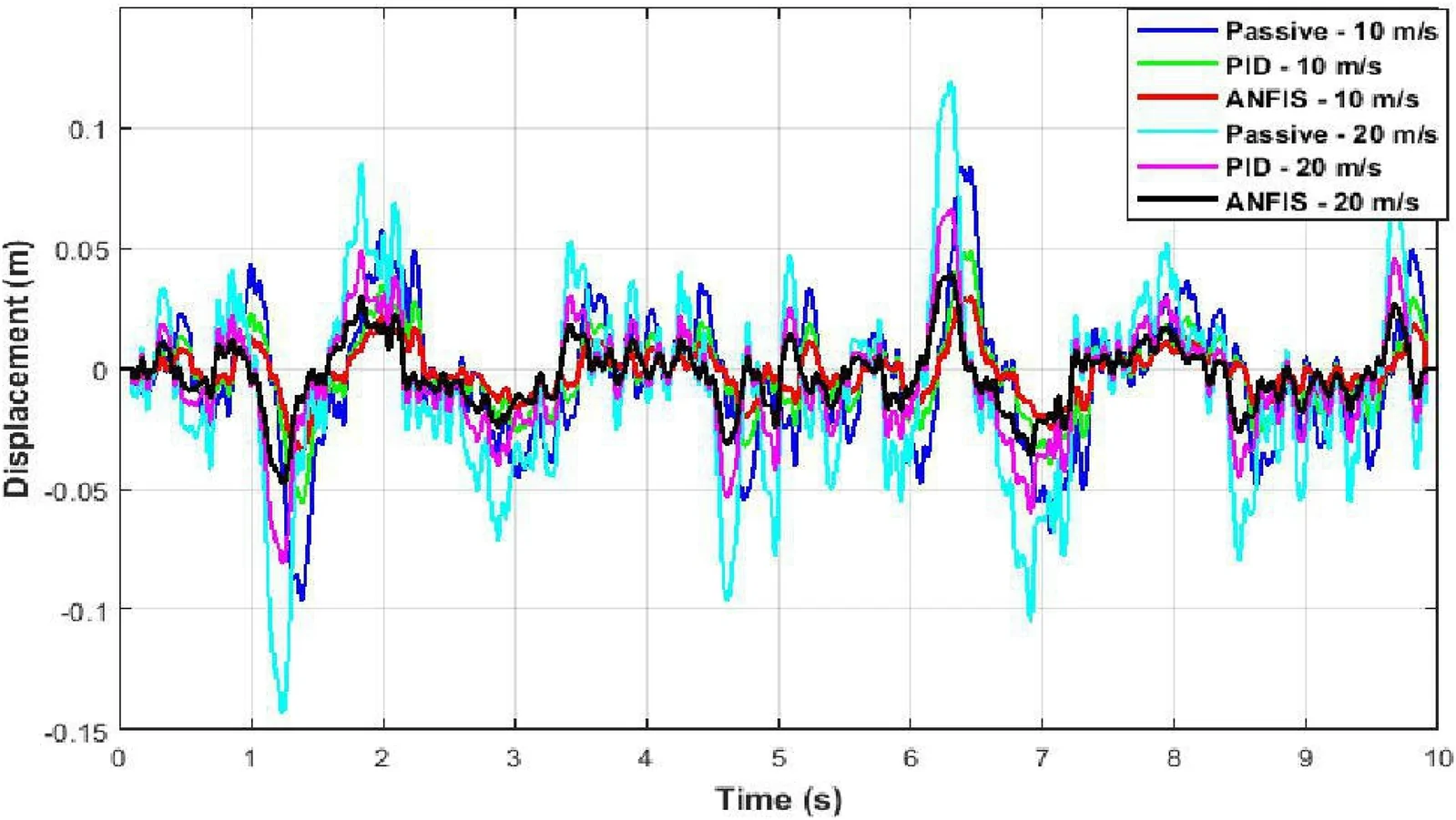
Suspension travel at rear wheel drive.

The controller was evaluated only with nominal model parameters and without explicit sensor-noise, actuator-delay, saturation, or parameter-variation tests. This is a significant limitation because uncertainty in sprung mass, tire stiffness, damping, and actuator dynamics can change the closed-loop response. Robust fuzzy-sliding-mode and disturbance-rejection methods have recently been proposed specifically to address uncertainty, measurement noise, and disturbance rejection [11]. The present ANFIS structure may tolerate some variation because its nonlinear mapping is learned over a range of operating conditions, but robustness cannot be inferred from the nominal simulations alone. Future work should conduct Monte Carlo studies with ±10–20% parameter perturbations, additive sensor noise, actuator delays, and force saturation, followed by hardware-in-the-loop and experimental vehicle testing.

## 4. Conclusions

The proposed ANFIS-controlled active suspension was evaluated against passive and PID-controlled suspension using a 4-DOF longitudinal half-car model under double-bump and ISO Type A and Type C random road profiles at 10 and 20 m/s. Under the double-bump input at 10 m/s, ANFIS reduced body vertical displacement by 80.6%, vertical acceleration by 67.9%, and front suspension travel by 86.2% relative to PSS; settling-time reductions for the principal body and pitch responses were approximately 39%. The corresponding improvements relative to PID were smaller but remained substantial, indicating that the learned nonlinear control mapping can improve transient vibration suppression beyond the tuned PID baseline. The random-road simulations also showed consistent reductions in RMS body motion and suspension travel for both Type A and Type C profiles at 10 and 20 m/s.

The physical interpretation is that ANFIS is most effective at reducing the amplitude of the dominant transient excursions, whereas settling time remains influenced by the inherent natural frequencies and damping of the coupled half-car system. The study also shows that the claimed benefits should be interpreted carefully: the ANFIS controller was trained from PID-generated data, the vehicle parameters were adopted from a published model rather than experimentally identified, formal statistical significance cannot be obtained from the deterministic simulations, actuator energy was not evaluated, and robustness to parameter variation, sensor noise, actuator delay, and saturation was not demonstrated. In addition, the use of a linear half-car model limits generalization to strongly nonlinear suspension behavior and full vehicle roll dynamics. The next stage of the research should therefore combine direct adaptive fuzzy learning, uncertainty/sensitivity analysis, frequency-weighted ISO 2631 evaluation, actuator energy assessment, and hardware-in-the-loop or vehicle-level experimental validation.

## Supporting information

S1 File Half-Car suspension ANFIS simulation dataset. (XLSX)
